# Targeted drug monitoring in oncology for personalized treatment with use of next generation analytics

**DOI:** 10.1007/s12672-025-03376-4

**Published:** 2025-08-11

**Authors:** Wei Li, Chaoling Wen, Bin Ye, Pranjal Gujarathi, Meghraj Suryawanshi, Kuldeep Vinchurkar, Imtiyaz Bagban, Sudarshan Singh, Opeyemi Joshua Olatunji

**Affiliations:** 1 Second People’s Hospital of Wuhu, Wuhu, Anhui China; 2 Anhui College of Traditional Chinese Medicine, Wuhu, 241000 Anhui China; 3 University of North Carolina at Chapel Hill, Chapel Hill, 27599 USA; 4 Department of Pharmacology, Vidhyadeep Institute of Pharmacy, Vidhyadeep University, Anita, Surat, 394110 Gujarat India; 5 Department of Pharmaceutics, Sandip Institute of Pharmaceutical Sciences (SIPS), Affiliated To Savitribai Phule Pune University (SPPU, Pune), Nashik, 422213 Maharashtra India; 6 Department of Pharmaceutics and Pharmaceutical Technology, Krishna School of Pharmacy and Research, Drs. Kiran and Pallavi Patel Global University (KPGU), Varnama, Vadodara, 391243 Gujarat India; 7 Office of Research Administration, Chiang Mai University, Chaing Mai, 50200 Thailand; 8 Faculty of Pharmacy, Chiang Mai University, Chaing Mai, 50200 Thailand; 9 African Genome Center, Mohammed VI Polytechnic University, Benguerir, Morocco

**Keywords:** Therapeutic drug monitoring, Cancer, Oncology, Immunoassay, Liquid-chromatography-mass spectroscopy

## Abstract

Therapeutic drug monitoring (TDM) is a clinical procedure aimed at maintaining plasma drug concentrations within a specific therapeutic range, thereby maximizing the safety and efficacy of pharmacological therapy. Conventional oncology strategies face challenges like non-specific toxicity, drug resistance, incomplete tumor eradication, high costs, and significant side effects that impact quality of life. Moreover, conventional therapy offers limited benefits in advanced stages, pose risks of secondary cancers and immune suppression, and lack personalization, highlighting the need for targeted, innovative approaches. In modern oncology, TDM has gained significant interest due to narrow therapeutic windows, significant inter-individual variability in pharmacokinetics, and the complexity of cancer pharmacotherapy. This study reviews the role of TDM in oncology with more emphasis on pharmacogenetic testing, immunoassays, and liquid-chromatography-mass spectroscopy (LC-MS/MS) techniques, highlighting its applications in optimizing the dose during immunotherapies, targeted therapies, and chemotherapeutics. Moreover, the review discusses the challenges and limitations associated with TDM in oncology, such as the requirement of robust clinical evidence, standardized practices, and integration with personalized medicine approaches. Emerging technologies, including AI and machine learning, are also considered for their potential to enhance TDM in oncology.

**Graphical abstract:**

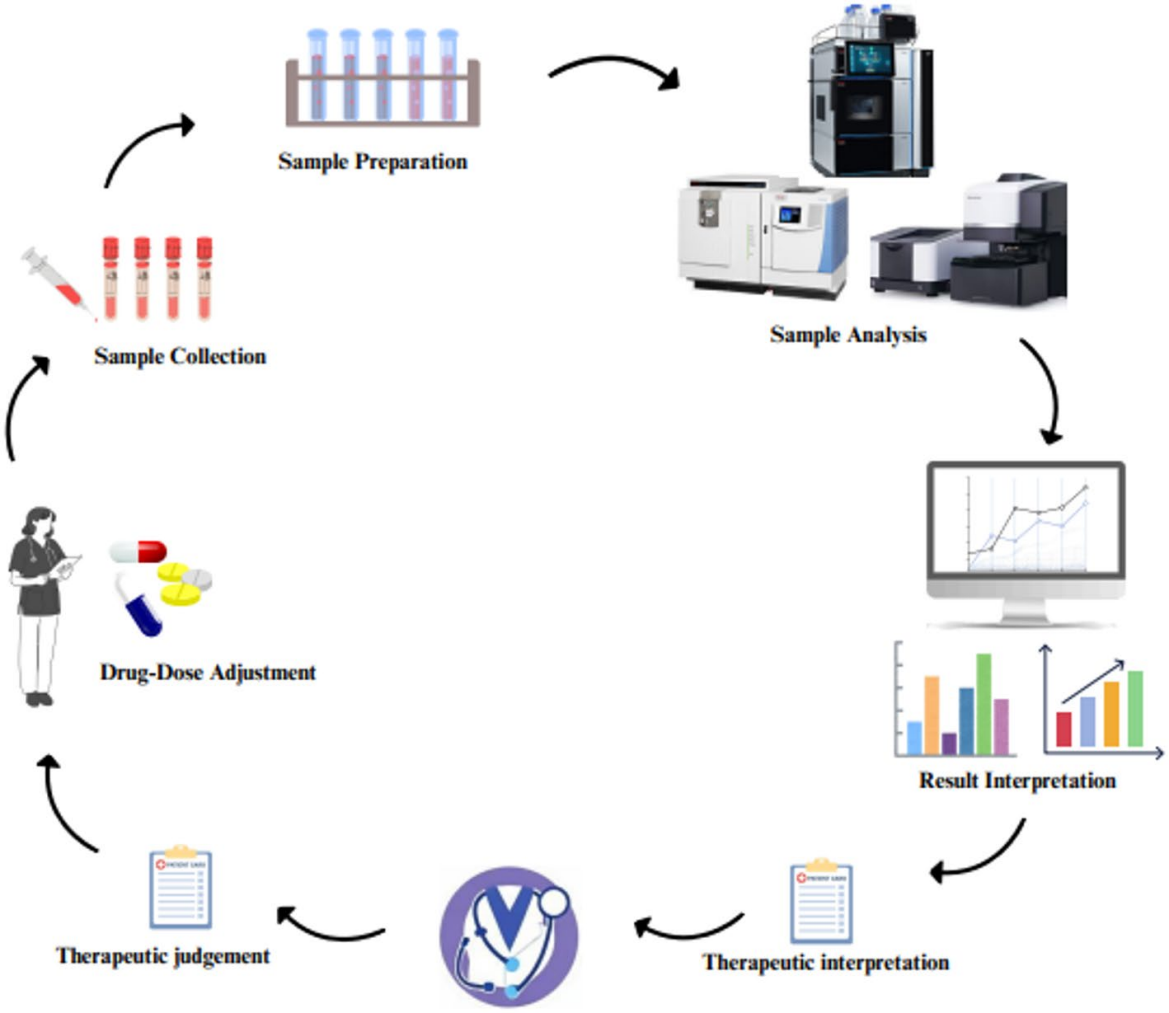

## Introduction

Therapeutic drug monitoring (TDM) in oncology has evolved from an unambiguous technique of measuring drug levels to a multidisciplinary approach for improving patient care through individualized dose adjustments [1]. The implementation of TDM has faced difficulties with cytotoxic medications, primarily due to obstacles in determining target concentrations and the complexities associated with combination therapies. However, it demonstrates potential for enhancing the efficacy of targeted therapies [2]. While TDM can address the substantial inter-individual pharmacokinetic (PK) variability influenced by factors such as pharmacogenetics, patient adherence, and drug interactions [2], some evidence-based targeted therapies, including imatinib and erlotinib, suggests that drug exposure correlates well with treatment response [2]. TDM may be particularly useful in situations such as a lack of therapeutic response, unexpected toxicities, or concerns about treatment adherence [3].

Despite wide inter-patient variability in drug concentrations, dosing based on body size remains the predominant approach [4]. Additionally, TDM plays a critical role in oncology by optimizing the use of anticancer agents, ensuring both efficacy and safety, and aligning with the principles of precision medicine. TDM clearly involves measuring systemic drug concentrations to individualize dosing for drugs with narrow therapeutic windows and significant PK variability. Thus, this approach is particularly valuable in preventing toxicities and maximizing therapeutic outcomes by tailoring treatments to patients-specific factors like metabolism, organ function, and genetic profiles.

TDM has shown promising results for certain anti-neoplastic drugs, such as imatinib, where the International Association of Therapeutic drug monitoring and Clinical Toxicology has developed consensus guidelines [5]. The use of TDM in cancer chemotherapy could ensure appropriate drug exposure and limit dose-related toxicities, addressing the challenges of dosing cytotoxic, targeted, and antibody-based biological anticancer drugs [6]. However, the widespread adoption of TDM in oncology faces obstacles, including logistical complexity, lack of clinical laboratories for drug concentration measurements, and insufficient evidence demonstrating improved clinical outcomes [7].

Cancer pharmacotherapy has evolved significantly over the past decades, moving from traditional approaches to more targeted therapies [8]. The field encompasses basic pharmacological principles, including PK and pharmacodynamics, which are crucial for individualizing patient treatment [9]. Recent progress in the field of cancer biology has resulted in the development of a variety of small molecules and biological agents, thereby broadening the range of treatment options accessible to oncologists [10]. While classic treatments strategies like surgery, radiotherapy, chemotherapy, and endocrine therapy remain important, and their mechanisms of action are now more acceptable [11]. The current focus is on molecularly targeted therapies, including antibodies, small molecules, and anti-angiogenic drug that are designed to exploit specific tumor characteristics [10, 11]. As the field progresses, the integration of biomarkers and a deeper understanding of drug interactions are crucial for optimizing cancer treatment strategies [11].

Cancer is major societal, public health, and economic problem in the 21st century, responsible for almost one in six deaths (16.8%) and one in four deaths (22.8%) from non-communicable diseases worldwide [12]. In addition, to being an important barrier to increasing life expectancy, cancer is associated with substantial societal and macroeconomic costs that vary in degree across cancer types, geography, and gender [13]. The current treatment approaches include surgery, radiotherapy, and chemotherapy, with personalized therapies showing promising results [14]. However, genomic and transcriptomic studies have driven recent advances in cancer pharmacotherapy, enabling more targeted treatments [15]. Molecular imaging techniques like Positron Emission Tomography (PET) and Magnetic Resonance Imaging (MRI) have transformed the landscape of drug development and personalized medicine by offering intricate, non-invasive, and real-time observations of the body’s physiological functions at a molecular scale. This advancement empowers researchers and healthcare professionals to accurately assess the effectiveness of medications, focus on particular disease locations, and customize treatment strategies according to the unique responses of individual patients, ultimately facilitating more efficient and targeted therapies, while minimizing the adverse effects [15]. Additionally, marine-derived pharmaceuticals have contributed significantly to cancer via chemotherapy, with several approved drugs and others in late-stage of clinical trials [16]. These include cytarabine, trabectedin, and eribulin, among others [16]. Despite progress, only about 10% of cancer patients achiev a complete cure or prolonged remission through chemotherapy alone, highlighting the ongoing need for improved therapeutic strategies [17].

Cancer is a global health concern, with an estimated 18.1 million new cases and 9.6 million deaths worldwide in 2018 [18]. Pulmonary, mammary, and colorectal malignancies rank among the most prevalent forms of cancer diagnosed worldwide [19]. Lung cancer is the leading cause of cancer-related deaths, followed by stomach and liver cancers [19]. Cancer incidence and mortality rates vary geographically and ethnically, influenced by factors such as age, gender, and economic development [19]. While cancer survival rates have improved in developed countries over the past 30 years, developing nations face challenges due to inadequate screening programs and delayed diagnoses [20]. The overall risk of developing cancer between ages 0 to 74 is 20.2%, with men’s is slghtly at higher risk, compared to women’s. Moreover, cancer is projected to become the first cause of death worldwide by 2060 [18–21]. Among the prevalent forms of cancer, lung, breast, colorectal, and prostate malignancies rank highest in terms of incidence rates. Notably, lung cancer accounts for the highest proportion of cancer-related mortalities. However, the incidence and mortality patterns exhibit significant geographic variations, with transitioned nations generally experiencing higher rates compared to transitioning countries [22].

**Fig. 1:**
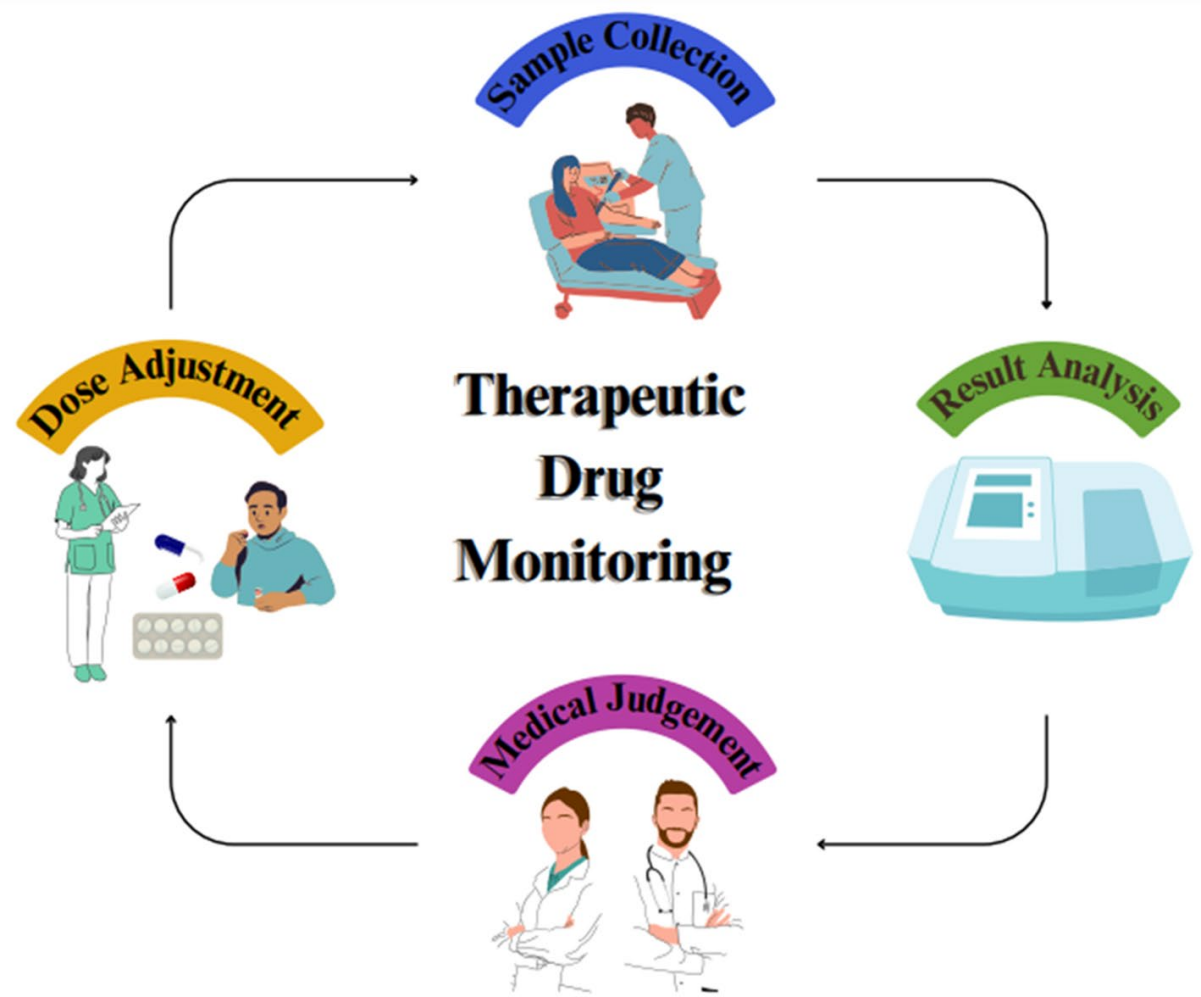
Overview of therapeutic drug monitoring application in oncology

TDM in oncology aims to optimize anticancer drug dosing by adjusting treatment based on individual patient factors and drug exposure. Despite the narrow therapeutic index and high PK variability of many anticancer agents, TDM is still uncommon in clinical practice [23]. Challenges include difficulties in establishing target concentration ranges, the use of combination therapies, and limited pharmacological trial data. However, TDM has proven beneficial for specific drugs like methotrexate, carboplatin, and busulfan, particularly in pediatric oncology. Nevertheless, advancements in PK, pharmacodynamics, and analytical techniques are addressing these barriers. As precision medicine continues to evolve, TDM is emerging as an essential strategy for improving treatment outcomes and minimizing adverse effects in cancer care. Thus, to advance TDM implementation, further research is required to define concentration-effect relationships, develop population PK/pharmacodynamics models, and conduct comparative trials of standard versus TDM-guided dosing. Ultimately, TDM in oncology aims to enhance treatment efficacy, minimize toxicity, and improve patient outcomes [23]. To further precision oncology, this review provides a thorough understanding of TDM’s potential to improve treatment outcomes and reduce side effects in cancer patients (Fig. 1).

### Principles of therapeutic drug monitoring

TDM is a clinical conduct to measure the drug concentration in biological samples periodically to adjust the drug dosages to individualize the treatment. Therefore, TDM draws a proper conclusion about drug concentration and dose adjustment by comparing the obtained concentration of the drug in a biological sample with its PK parameters [24]. The International Association for Therapeutic drug monitoring and Clinical Toxicology defined “TDM is the quantification executed based on laboratory parameters that on proper interpretation directly influence prescribing procedures. Generally, the quantification of prescribed drugs in biological samples can be also performed for endogenous agents prescribed as replacement therapy in a person who has a physiological or pathological deficiency of that agent [25]. The whole process of TDM includes a series of steps starting from the diagnosis of the disease to the optimization of drug dose [26]. Thus, TDM aims to optimize the therapeutic outcome in an individual by changing the drug dose to improve efficacy and reduce toxicity or adverse drug reactions. Besides, TDM is also used to monitor a patient’s compliance with a drug regimen and to point out possible drug-drug or food-drug interactions [27].

### Pharmacological characteristics of drug candidates require therapeutic drug monitoring

**Fig. 2:**
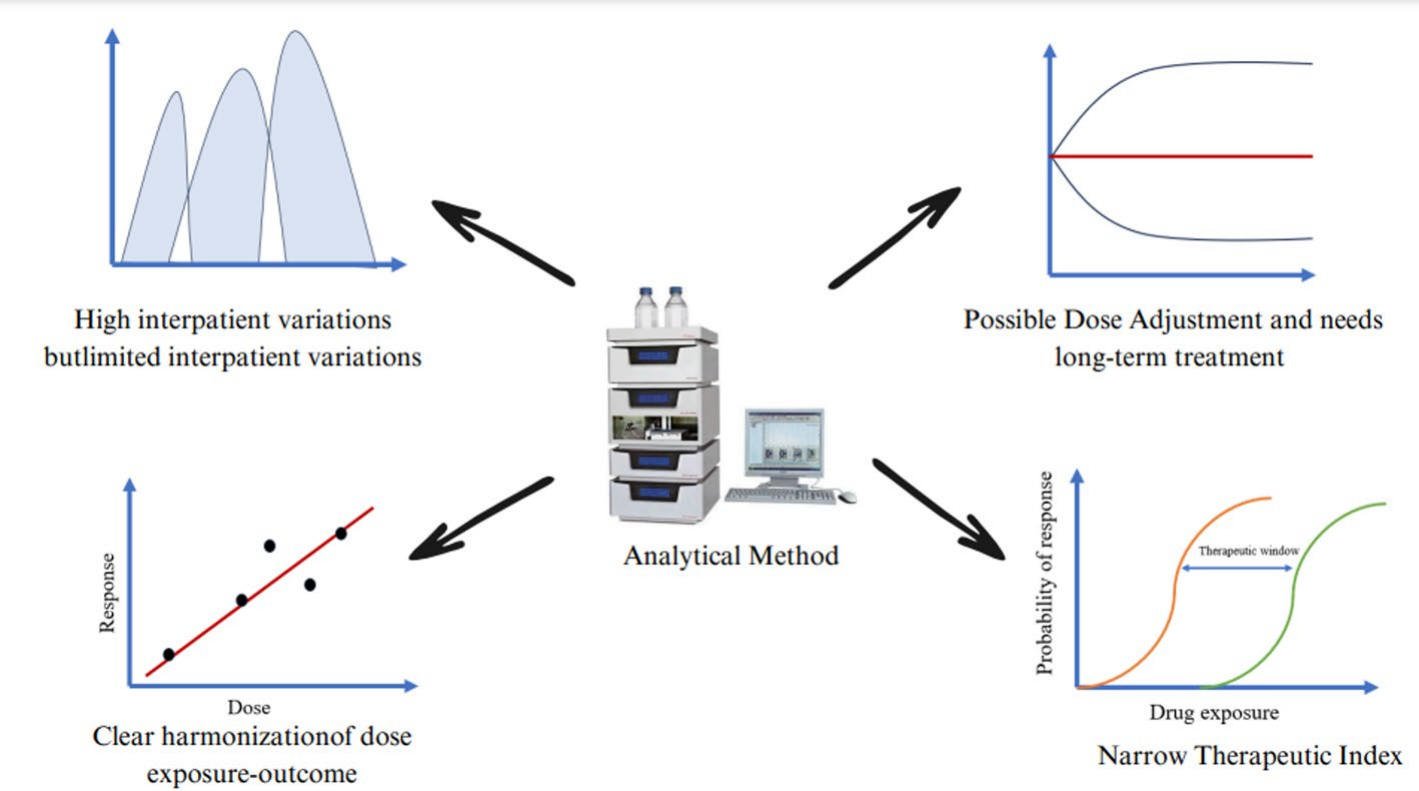
Important characteristics of the candidate need therapeutic drug monitoring

TDM is not generally required for every drug candidate, every patient, and every disease. The important characteristics of drug a candidate is illustrated in Fig. 2. Several authors described the properties of TDM drug candidates [25] that have been recapitulated as follows:

- Unavailability of clinically defined parameters that permit the adjustment of dose.
- Uncertain correlation between the dose and clinical outcome. Example, a defined dose of the drug may give the intended pharmacological action in one individual, but an exact similar dose of the same drug may lead to toxicity in another individual.
- The consequence of drug toxicity can be hospitalization, irreversible organ damage, and sometimes death.
- There is a significant difference among the patient’s PK, which is unable to predict easily depending on individual patient characteristics.
- The drug required to exhibit stable PK properties, limited inter-subject variability, make a TDM measurement representative of the patient’s regular exposure level.
- The narrow therapeutic margin concerning inter-subject uneven PK prohibits the utilization of high doses in all patients to confirm overall efficacy.

### Pharmacokinetics

The direct or indirect inter-linkage of a drug to its specific receptor and alteration in the physiological mechanism controlling the function leads to the pharmacological effect of the drug. Various studies have claimed that there is a direct correlation between the pharmacological action of various drug and their serum concentration. Thus, PK studies have established that local variations in drug levels at receptor sites or within tissues that correlate directly to fluctuations in blood drug concentrations over time. Additionally, during the post-absorptive and post-distributive phases of the time course, this principle referred to as the degree of kinetic homogeneity applies universally to all PK models. As it provides the foundation for all therapeutic and toxic concentration reference standards, the validity of kinetic homogeneity is a pivotal consideration in TDM [23, 28, 29].

### Absorption

Anticancer chemotherapeutics are generally administered by intravenous and oral routes. The intravenously administered drugs are quick and completely bioavailable due to direct availability into the systemic circulation [23]. Bioavailability generally high for soluble and stable drug that are administered orally and hence absorbed instantly and efficiently into the systemic circulation from the gut [29]. Among the existing approved oral anticancer drugs, about 65% exhibt low or poor water solubility and thus they do not achieve their potential therapeutic outcomes with maximum efficacy and minimum toxicity [30]. To be a therapeutic benefit, the drug administered by oral dosing and its active metabolites should demonstrate an effective systematic absorption when the substances pass through the gastrointestinal tract [29]. Oral formulations generally provide the necessary pharmacological effects only when bioavailability reaches this critical threshold [23].

The absorption process relies on the drug dissociating from its dosage form, dissolving gastrointestinal fluid, and its diffusion across the biological membrane, subsequently into the bloodstream. Moreover, the orally administered drug undergoes first-pass metabolism after entering into the systemic circulation. Thus, oral drugs possess higher PK variability than intravenous [23]. Intravenous injection studies of etoposide, an epipodophyllotoxin used in testicular and lung cancers provide a particularly salient example of this interpatient PK variability. Intravenously when etoposide was administered, the study showed a coefficient of variation and 28% fr area under curve (AUC) following oral. For testicular cancers, it is preferable to administer etoposide intravenously rather than orally in patients due to potentially subtherapeutic drug levels distress [31]. Administration by the intravenous route minimizes variability, thereby helping to maintain appropriate drug levels in patients [31].

### Distribution

As soon as the drug reaches to blood, it moves to various organs and tissues via various transport channels and interacts with proteins such as albumin, α- 1-acid glycoproteins (AAG), etc [32]. The drug distribution depends on the equilibrium between protein-bound and free drugs because only the free form of the drug can cross the cell membrane and reflect the pharmacological action. The binding of drugs to proteins significantly affect the distribution of a drug through the body. Therefore, patients with different plasma protein concentrations demonstrate PK differences in this pathway [32]. Serum albumin levels, for instance, are typically decreased in conditions such as mal-nutrition and liver metastases. This decrease in albumin would then lead to higher levels of freely available drugs in the systemic circulation [32]. On the other hand, inflammatory conditions may increase drug-protein binding due to acute phase proteins such as α-1-acid glycoprotein are produced [23, 32]. Because binding prevents the drug from being active, there are fewer pharmacologically free drugs to measure the therapeutic effect [32].

### Metabolism

The liver is the primary site of drug metabolism and it also has an important role in PK alterations [31]. The Organic anion-transporting polypeptide protein family is responsible for the movement of drugs from blood to hepatocytes. After reaching the liver drug undergoes Phase I and Phase II biotransformation reactions. PK variability the inter-individual depends on the polymorphic genes of transporter proteins and Phase I and Phase II enzymes [31]. Moreover, in Phase I biotransformation reaction drug undergoes the oxidation, reduction, hydrolysis, cyclization, and de-cyclization reactions. The cytochrome P 450 enzymes are mainly responsible for the oxidation in the Phase I reaction [33]. CYP1A, CYP2B, CYP2C, CYP3A isoforms of CYP450 are responsible for oxidation of anticancer agents. Mainly, detoxification reactions are involved in the Phase-II biotransformation process, that includes glucuronidation, sulphation, and methylation [33]. Whereas the Phase-II reaction’s product increases metabolite’s molecular weight and converts it into an inactive form [34].

### Elimination

Most of the anticancer drugs are excreted through the biliary tract and kidneys. Very little chemotherapeutic is excreted through sweat, lungs, skin, and tears. The biliary excretion of amphipathic and lipid soluble chemotherapeutic generally occurs through ATP-Binding cassettes transporters [35]. Cisplatin and Methotrexate (MTX) are primarily eliminated through the renal route. The sub-therapeutic concentration of MTX was observed when it was administered with bicarbonate, as the bicarbonate transform the pH to basic and promotes the MTX renal clearance [36].

### Other factors of Pharmacokinetic alterations

Age and poly-pharmacy are equally responsible for the PK variability. The absorption of medications in elderly patients can be influenced by age-related physiological changes, such as decreased gastric acid secretion, reduced intracellular water content, and diminished splanchnic blood flow. Additionally, the increased proportion of body fat and decreased plasma albumin levels associated with aging can lead to alterations in drug distribution within the body [37, 38]. Increased body fat, and decreased plasma albumin with age lead to alterations in drug distribution. 20–50% of lier mass is reduced the activity of CYP-450 isoenzymes is also decreased as well as in geriatric patients of 80 years which ultimately affects the process of metabolism [39, 40]. Moreover, the hepatic blood flow and glomerular filtration rate is reduced in the aged patient, affecting the drug’s clearance [40].

The effects on the four key components of drug disposition are also influenced by co-administration with drugs [41]. The implications of polypharmacy are numerous: from how drugs are absorbed, distributed, metabolized, and finally excreted. There are also some examples of polypharmacy that can influence PK, where tamoxifen used for breast cancer chemotherapeutics is likely co-administered with selective serotonin reuptake inhibitor antidepressant drugs [41]. In a study on 80 patients with breast cancer, co-administration of the selective serotonin-reuptake inhibitor paroxetine reduced circulating levels (mean reduction, 56%) of edoxifen, an active metabolite product from tamoxifen [41].

### Analytical techniques used in therapeutic drug monitoring

TDM involves the quantification of the drug and its metabolite in physiological fluids like plasma, serum, and urine at various time intervals and various stages of treatment using modern analytical techniques [42]. The pharmacological or toxic effects of drugs are generally determined by the estimation of drug concentration in blood/plasma for most of systemic therapies. The efficacy of drug is more reflects by the intracellular drug concentration than the plasma or whole blood [42]. The human peripheral blood mononuclear cells have also been used in TDM for the sample matrix. In addition, the dried blood spots, offer dual benefits of being minimally invasive and requiring minute blood volumes, and have also been rarely utilized for drug concentration determination. On the other hand, urine, less frequently hair, and various tissues are rarely employed for TDM [42].

For much diluted samples the TDM strategies are highly dependent on precise and unbiased analytical techniques. Besides, automation, high-throughput instruments, robustness, and cost-effectiveness are the requisites for choosing a suitable analytical technique [43]. Immunoassays are widely used in TDM; however, the immunoassay techniques can encounter non-specific interferences from related compounds, metabolites, or effects of physiological fluids. Nowadays, high-quality analytical methods such as liquid chromatography (LC) combined with ultra-violet (UV), fluorescence detection, or Mass spectroscopy (MS) detectors are mainly used in TDM as these techniques are more reliable and highly sensitive [43]. The potential of LC to segregate individual components from other drugs and metabolites present in the physiological fluids, in combination with chosen detection techniques methods, offers high sensitivity and specificity. To obtain relevant drug measurements and abstain from the interference of physiological fluids the sample preparation, column technology, selection of internal standards, and detection parameters are immensely important [43].

The choice of the robust analytical conditions during the validation ought to prove the selectivity of the column to the analyte, besides optimization of the analyte; this should also produce clear and distinct chromatograms free from isobaric interferences [43]. Analyte contents are detectable following administration for quite a long time; thus sensitivity is not an issue of contention when the analytical procedure is being performed on biological fluids. The ultraviolet detector can be a valuable tool if the selectivity assessment takes into account potential interactions with co-administered compounds and the sample matrix [43]. During the recent years liquid chromatography associated with mass spectrometry (LCMS) or multiple mass spectrometry’s (LC-MS/MS) has shown higher application for drug analysis and at present is widely accepted as an analytical standard in TDM. LCMS and tandem mass spectrometry techniques remain crucial for determining various drug classes when alternative methods are unsuitable [43].

Furthermore, analytical validation in TDM involves several critical parameters to ensure the accuracy and reliability of drug concentration measurements. Whereas sensitivity and specificity are essential for distinguishing between drug and their metabolites or endogenous substances, ensuring that the method can accurately detect and quantify the target drug without interference. The limit of detection is the lowest concentration that can be detected, while the limit of quantification is the lowest concentration that can be quantified with acceptable precision and accuracy. For TDM, achieving a low limit of quantification is crucial for accurately measuring drug levels near the therapeutic range. Additionally, validation includes assessing calibration models, accuracy, precision, dilution integrity, matrix effects, and stability to ensure that the analytical method provides reliable data for clinical decision-making. These parameters are often validated according to guidelines from regulatory bodies like FDA and EMA, although TDM may require more streamlined validation processes due to its specific clinical application. Additionally these techniques offer superior sensitivity and selectivity compared to other analytical approaches, making them invaluable tools in TDM.

### High-performance liquid chromatography-ultraviolet visible spectroscopy

TDM system utilizes HPLC to determine polar and nonpolar metabolites. HPLC is cost-effective and the most widely used analytical technique for qualitative and quantitative estimation of the drug and its metabolite. Anticancer agents are comparatively administered at high concentrations; therefore, the high-performance liquid chromatography-ultraviolet visible spectroscopy (HPLC-UV) method determines them effectively [44]. A group of researchers determined the androgen receptor signalling pathway inhibitor and competitive inhibitor of dihydrotestosterone–enzalutamide, in the plasma of patients (*n* = 16) suffering from metastatic castration-resistant prostate cancer by the HPLC-UV method [42, 44, 45].

The introduction of tyrosine kinase inhibitors revolutionized cancer treatment by transforming some previously terminal malignancies into chronic conditions. Tyrosine kinase inhibitors are also used to treat other ailments, such as autoimmune diseases [46]. After oral administration, intricate processes involved in drug absorption and metabolism result in substantial inter- and intra-patient variability of tyrosine kinase inhibitor plasma levels. Consequently, given the high variability of tyrosine kinase inhibitor concentrations and their therapeutic and toxic effects, patient drug levels should be monitored [46]. To quantify ponatinib, a third-generation tyrosine kinase inhibitor employed to optimally inhibit native and mutated BCR-ABL, including the gatekeeper mutant T315I, an HPLC-UV method was developed [45].

### High performance liquid chromatography-fluorescence detector

Fluorescence detection can provide distinct advantages in sensitivity and selectivity, compared to absorbance detection, and it can prove useful for the analysis of various pharmaceuticals in biological samples. In some instances, the fluorescence detector allows for nearly 30 times greater sensitivity than the UV detector, which is particularly crucial for examining biological samples where analyte concentrations are low. At times, a fluorescence detector has been utilized for the quantification of anticancer drugs either without or with chemical derivatization [47]. The high performance liquid chromatography-fluorescence detector (HPLC-FLD) method for epirubicin, which act by intercalation of DNA strands was developed and validated by Trader and co-workers [47].

For the determination of analytes that lack strong chromophores or fluorophores, derivatization is required before analysis via HPLC-UV/FLD. As an example, L-asparaginase, a therapy used to treat acute lymphoblastic leukemia in children, can be quantified in patient serum samples through HPLC-FLD following a derivatization step [48]. This pre-treatment reaction ensures the addition of fluorogenic or chromogenic moieties, allowing for selective detection and measurement of the target analyte that otherwise would not significantly interact with the detection system. Derivatization is thus a useful sample preparation technique when HPLC-UV/FLD is the preferred analytical platform but the compound of interest is not inherently UV or fluorescence-active [48].

### Liquid chromatography-mass spectroscopy

Liquid chromatography coupled with mass spectroscopy and LC-MS/MS techniques with the use of ESI as a source of ions as well as triple quadrupole mass analyser are widely used as anticancer drug and its metabolite determination in TDM of anticancer [49]. The samples for LC-MS/MS are prepared by a solid phase extraction, liquid-liquid extraction, protein precipitation, filtration, derivatization, dilution, concentration, and sometimes enzymatic digestion depending on the sample matrix and analyte of interest. The LC-MS/MS method for simultaneous tracking of 11 tyrosine kinase inhibitors such as, imatinib, dasatinib, nilotinib, bosutinib, ponatinib, ruxolitinib, brutinib, filgotinib, tofacitinib, baricitinib, and gefitinib in human plasma was developed by Koller and co-workers [49]. For the treatment of advanced renal cell cancer, imatinib-resistant or intolerant gastrointestinal stromal tumors, and pancreatic neuroendocrine cancers, sunitinib is used [50]. Most anticancer drug analyzes utilize C-18 or occasionally C-8 alkyl-bonded stationary phases for chromatographic columns. Few reports examined alternative columns like strong cation exchange. HPLC-UV and LC-MS/MS methods have been developed to analyze the tyrosine kinase inhibitors Dasatinib and Imatinib in plasma or serum as indicated in a recent study by Marangon and co-worker [51].

### High-performance liquid chromatography-ultraviolet visible spectroscopy and liquid chromatography-mass spectroscopy

Certain researchers have proposed employing both HPLC-UV and LC-MS techniques for quantifying anticancer therapeutics in biological specimens [50]. MTX, a folate antagonist that acts to inhibit dihydrofolate reductase and thereby prevent malignant cell division, has been evaluated utilizing LC-MS/MS within human plasma samples. The quantification detection was performed in a triple-quadruple tandem mass spectrometer under positive mode monitoring the mass transition of: *m/z* 455.3 to 308.3 for MTX and *m/z* 136.1 to 94.4 for internal standard tested. Moreover, the intra- and inter-day precisions were < 5.2%, he accuracy varied from − 4.1 to 4.5%, hereas the recovery was > 945. This result suggested that LC-MS/MS methods was in agreement with the existing HPLC-UV method with Passing-Bablok regression and Bland-Altman difference plot analysis. The study further suggested that validated LC-MS/MS can be successfully applied to the routine TDM of MTX in clinical settings [50]. There are a limited number of reports on analytical examinations of anticancer medications that have employed other types of columns, such as strong cation exchange, HPLC-UV, and LC-MS/MS methods have been developed for the analysis of the tyrosine kinase inhibitors Dasatinib and Imatinib in plasma or serum samples [52]. Various other examples of anticancer and its metabolite identified by chromatographic techniques are presented in Table 1.

**Table 1:** Representative anticancer and its metabolite identified by chromatographic techniques

Name of drug	Physiological fluid	Technique used	Mobile phase	Stationary phase	Sample preparation	References
Mitotane	Plasma	LC-UV	Acetonitrile (ACN), Potassium hydrogen phosphate, orthophosphoric acid and tetramethylammonium	C-8 column	Solvent-methanol, protein precipitation	[53]
Mitotane and (1,1-(o, p’-dichlorodiphenyl)-2,2 dichloroethene) (DDE) and (1,1-(o, p’-dichlorodiphenyl) acetic acid) (DDA)	Plasma	HPLC-UV	mitotane and DDE, acetonitrile, methanol, and water Mitonitate and DDE ACN, methanol-water, triethylamine	C-18 column	Liquid-liquid extraction (LLE) using acetone for mitotane and DDE LLE with methanol and acetonitrile mixture for DDA	[54]
5-fluorouracil and its metabolite	Plasma	LC-MS/MS	Methanol, water, ammonium acetate and formic acid	C-18 column	LLE using ethyl acetate and 2-propranolol	[55]
Tamoxifen	Plasma	HPLC-PDA	ACN, water, and formic acid	C-18 column	LLE using hexane and n-propranolol followed by re-extraction with phosphoric acid	[56]
Erlotinib, gefitinib, imatinib, dasatinib, lapatinib, nilotinib, sorafenib, sunitinib	Plasma	LC-TMS	Methanol, water, and ammonium hydroxide	C-18 column	Protein precipitation using ACN	[57]
Imatinib, dasatinib, nilotinib	Plasma	HPLC-UV	ACN, water, triethanolamine	C-18 column	LLE using hexane and n-propranolol	[58]
Imatinib, dasatinib, nilotinib	Mononuclear cells of blood	HPLC-MS	ACN, Water, formic acid	C-18 column	LLE using ACN and methanol	[59]
Ibrutinmib	Plasma	HPLC	ACN, water, and monopotassium phosphate	C-18 column	Solid phase Extraction (SPE) on Oasis HLB cartridges	[60]
1-β-d-Arabinofuranosylcystosine 1-β-d-Arabinofuranosyluracil	Plasma & Urine	LC-TMS	ACN and water or methanol, water, and formic acid	C-18 column	Protein precipitation using acetonitrile	[61]
Hydroxyurea	Plasma	HPLC-UV	ACN, Water, and ammonium acetate	C-18 column	Protein precipitation using methanol	[62]
Rituximab	Serum	MS	ACN, 2-propranolol, water, and formic acid	C-3 column	Vortexing	[63]
Methotrexate	Serum	HPLC	ACN and sodium acetate buffer	C-18 column	SPE on SPE LC-Ph cartridges with phenyl stationary phase Cartridges preconditioning- Methanol followed by water	[64]
Methotrexate	Plasma	HPLC-UV	ACN, water, and sodium acetate, acetic acid	C-18 Column	Protein precipitation using ACN, followed by LLE using chloroform and methylene chloride	[65]

### Surface enhanced Raman spectroscopy

**Fig. 3:**
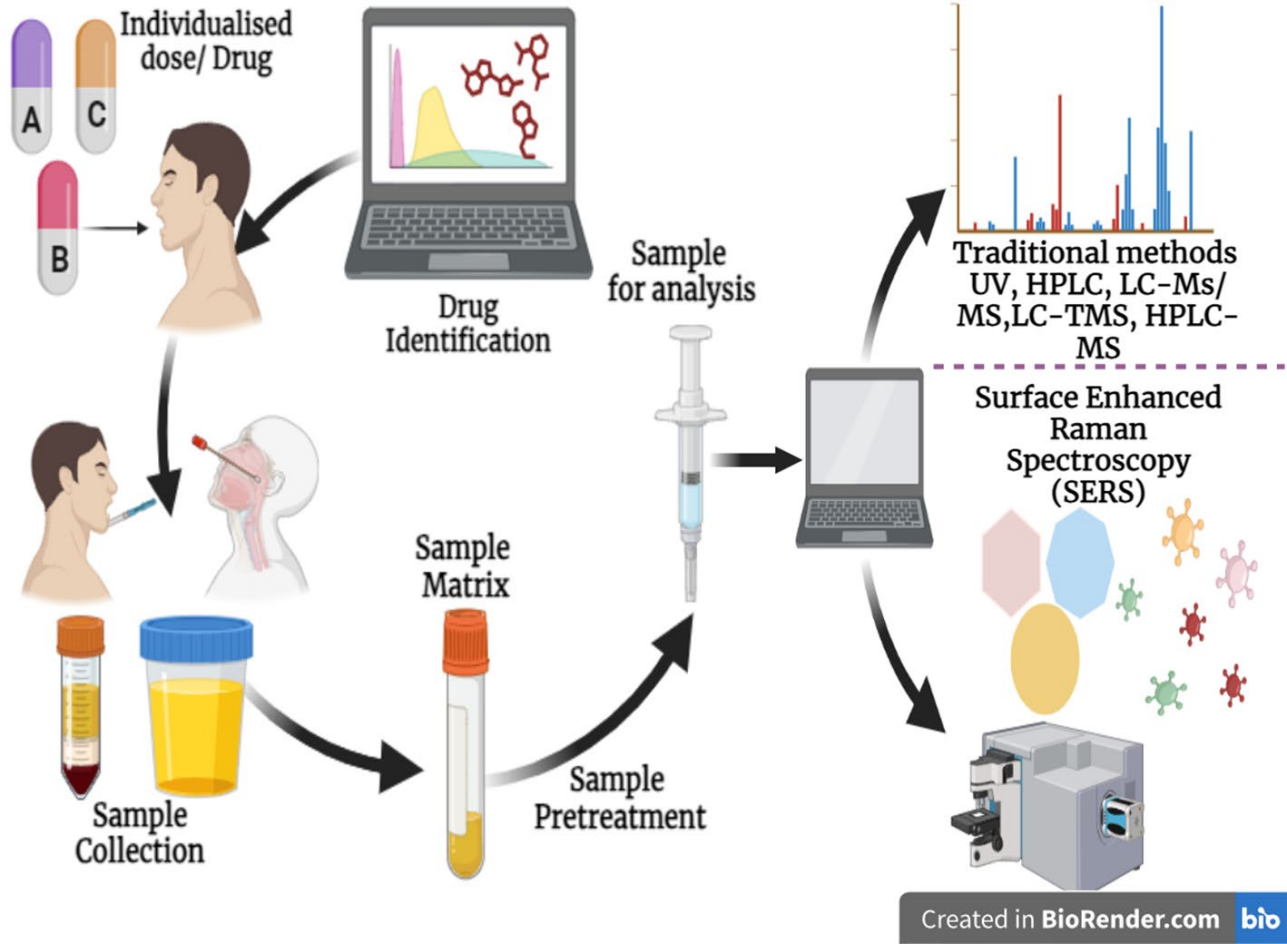
Traditional methods and SERS technique in therapeutic drug monitoring

Surface Enhanced Raman Spectroscopy (SERS) is a label-free technique that enables the rapid monitoring of substances at low concentrations in biological matrices. This key advantage makes SERS an attractive tool for developing point-of-care tests suited for TDM of drugs with narrow therapeutic windows, such as chemotherapeutics, immune-suppressants, and various anticonvulsants [66]. In traditional analytical techniques sample is directly used for analysis while in SERS after sample preparation the sample need to couple with SERS-substrate Fig. 3.

SERS is also well-suited for TDM as, in principle; quantitative analysis of drugs in bodily fluids could be accomplished within a few minutes and with comparable or smaller errors than routine TDM methods. While SERS shares the challenge of surface fouling with other methods (e.g., SPR, QCM), the higher level of information present in SERS spectra facilitates leveraging the full potential of multivariate data analysis to limit interference from nonspecific binding [67]. Additionally, portable Raman spectrometers are now available that are robust, compact, and easy to use; thus, they could be routinely employed in clinical settings by non-specialized operators [68]. SERS for TDM of various categories as chemotherapeutic agents presented in Table 2.

**Table 2:** Surface enhanced Raman spectroscopy analysis of various anticancer drugs

Drug	Sample	SERS substrate	Laser line (nm)	References
5-FU	Saliva	SERS-active capillaries	785	[69]
MTX	Human Serum	Gold colloid on paper	785	[70]
6-mercaptopurins (6-MP)	Water	β-cyclodextrin gold nanoparticles	785	[71]
6-MP	Britton-Robinsons buffer	Graphene oxide-gold nanoparticles hybrid	532	[72]
6-MP	Whole Blood	Silicon-gold nanoparticles needles	785	[73]
Paclitaxel	Dimethyl sulphoxide	Gold nanocylinder UV-Nanoimprint Lithography	633	[74]
Paclitaxel	Human serum albumin	Gold colloid	532	[75]
Paclitaxel	Blood plasma	Gold-polystyrene beads	785	[76]
Imatinib	Water	Plasmonic Nanodome array	785	[77]
Imatinib	Plasma	Gold on glass with aluminium	785	[77]
Doxorubicin	Plasma-Bovin	Gold colloid	488	[78]
Mitoxantrone	Serum of cancer patient	Gold colloid FLOW	514, 633	[79]
Mitoxantrone	Water	Plasmonic nano dome array	785	[80]
Methotrexate	Potassium hydroxide	Silver colloid FLOW CELL	514, 785	[81]
Methotrexate	Bovin serum albumin -phosphate buffer saline	Sandwich substrate	532	[82]
Methotrexate	Water	Gold-graphene	785	[83]
Irinotecan	Human serum albumin-phosphate buffer saline	Silver and gold colloid on thin layer chromatographic plate	514, 785	[84]

### Pros and cons of analytical methods

Generally, Immunoassays are commonly employed methods due to their convenience and rapidity. However, they exhibit limitations including interference from matrix components, drug metabolites, structurally similar drugs, and endogenous compounds. Additionally, immunoassays are not developed for all drugs, which are presently monitored [66]. Conversely, reference methods such as LCMS are in some situations still viewed as the gold standard for TDM in clinical laboratories given their enhanced analytical robustness and relative freedom from interference [66]. Nonetheless, analysis through these techniques is time-intensive and laborious owing to the extensive sample preparation mandated and large sample volumes necessary to process bio-fluids [66].

TDM using chromatographic, mass spectrometric, and immunoassay techniques often requires expensive infrastructure that may not be available in smaller hospitals. Recently proposed alternative TDM platforms such as surface Plasmon resonance localized surface Plasmon resonance, electrochemical sensors, and quartz crystal microbalance aim to provide rapid results with limited infrastructure and sample preparation requirements. However, these methods are susceptible to bio-fouling issues during measurement, preventing their routine use for large-scale testing [85, 86]. Non-specific binding of unwanted proteins and other molecules to device-sensing interfaces can easily foul biosensors’ active surfaces, generating overwhelming background signals and preventing detection of target drugs. Despite recent research efforts to mitigate fouling, it remains an unresolved issue. Therefore, new techniques that are less costly and faster than current reference methods yet can be regularly and easily used in hospitals are still needed [85]. The summary of advantages and disadvantages of various analytical techniques are discussed in Table 3.

**Table 3:** Advantages and disadvantages of analytical techniques used in therapeutic drug monitoring

Analytical methods	Advantages	Disadvantages
Chromatographic techniques: GC-MS/MS, LC-MS/MS	• Gold standard method • Robust with great sensitivity and sensibility • Comparatively less/ no interferences • Decreased drug class/metabolites cross-reaction	• Required greater time • Laboratory-developed method • Variation between results of two laboratories • Affected by physiological solutions • Expensive installation, personnel training and method validation•
Immunoassay Antibody conjugated magnetic immunoassay, cloned enzyme donor immunoassay, chemiluminescent microparticle immunoassay, enzyme-linked immunosorbent assay, enzyme multiplied immunoassay technique, fluorescence polarization immunoassay. microparticle enzyme immunoassay, particle-enhanced turbidimetric inhibition immunoassay	• Very small amount of sample required • Run on automated, steady, and random access systems • Sample clean-up is not required • Ability of multiplexing\	• Quantification of the analyte required several steps • Decreased specificity and sensitivity • Antibody cross-reactivity • Disturbances from bilirubin, haemoglobin, high lipid amount, very high and low protein concentration, various drug and its metabolites
SERS-techniques	• No sample preparation required • Rapid measurement • Portable Raman spectrophotometer available	• Frequent and high relative standard deviation of substrates • Each drug demands method optimization and validation

### Pharmacogenetics

Pharmacogenetics refers to the study of how an individual’s genome impacts differences in drug efficacy and toxicity between individuals. The metabolism of foreign substances is often categorized into three phases: modification (phase I), conjugation (phase II), and elimination. Phase-I drug-metabolizing enzymes, especially members of the CYP450 family, are responsible for the oxidation, reduction, and hydrolysis of medications [87]. Phase II drug-metabolizing enzymes, such as glutathione S-transferases (GSTs) and uridine diphosphate glucuronosyltransferases (UGTs), mainly inactivate or activate medications through conjugation reactions. Variations in these enzymes have frequently been shown to influence the PK of several anticancer drugs [87].

Glutathione S-Transferase α-1 plays an important role in the PK of busulfan, melphalan, and chlorambucil. Paediatric studies have shown that the GSTA1*B variant reduces busulfan clearance by up to 30% [88]. Whereas the clearance of thiotepa was predominantly affected by the GSTP1 C341T polymorphism that had a frequency of 9.3% i adult hematopoietic stem cell transplantation patients [88].

Other Phase-I enzymes include keto-reductase, aldehyde dehydrogenase, carboxylesterases, and dihydropyrimidine dehydrogenase (DPD), which is one of the best-characterized functional variants affecting anticancer agents. 5-FU and its oral prodrug capecitabine are two of the most frequently prescribed chemotherapeutic drugs. Both drugs require enzymatic activation of fluoropyrimidine nucleotides to exert their cytotoxic effects. Degradation of 5-FU plays a significant role as more than 80% of 5-FU is catabolized by DPD [88]. Reduced DPD activity results in increased exposure to 5-FU, which can lead to severe toxicity and even fatality at times. Compelling evidence demonstrates that patients with a partial or complete DPD deficiency demonstrate a reduced capacity to degrade 5-FU and are at risk of developing severe 5-FU-associated toxicity. Genetic testing for DPD activity is now recommended at many institutions before initiating 5-FU and capecitabine [88]. Moreover, polymorphisms in genes encoding drug efflux transporters, such as P-glycoprotein influence the uptake and excretion of anticancer drugs.

### Application of therapeutic drug monitoring in some common anticancer drugs

#### Methotrexate

Methotrexate is a drug that blocks the growth of cells by interfering with Deoxyribonucleic acid (DNA), Ribonucleic acid (RNA), and protein production. It’s commonly used to treat leukemias and lymphomas. Studies have shown that customizing MTX doses for each paediatric leukemia patient leads to better outcomes compared to giving everyone the same fixed dose [89]. MTX blood levels correlate with drug efficacy and adverse effects. The impact varies based on low or high dosing. Monitoring drug levels aids in optimizing patient-specific dosing [90]. MTX’s distinct feature is its reversible action through folinic acid (leucovorin) administration. Moreover, monitoring plasma levels enables personalized leucovorin rescue therapy based on the patient’s PK profile. When MTX levels become toxic, leucovorin treatment can ‘rescue’ bone marrow and intestinal epithelial cells by allowing DNA replication to resume.

TDM is recommended for high-dose Methotrexate (HDMTX) therapy due to significant variability in systemic concentration levels among and within patients [91–93]. Prior studies indicated substantial variability in patient responses to HDMTX. Additionally, an inter-patient variability has been reported at 52%, whie intra-patient variability of 48%. Thi heterogeneity manifests in the wide range of nephrotoxicity incidence, from 1.8% to 1.7%, obsrved across clinical trials. Monitoring HDMTX levels enables clinicians to optimize efficacy and safety outcomes for individual patients [91]. MTX elimination can be impacted by several drug interactions, including non-steroidal anti-inflammatory drugs, ciprofloxacin, probenecid, sulfamethoxazole, trimethoprim, amiodarone, tyrosine kinase inhibitors, and proton pump inhibitors. Additionally, certain medical conditions like down syndrome can also affect MTX clearance too [94]. TDM and optimized dosing of MTX and leucovorin maintain MTX concentrations within a therapeutic range. High-dose of MTX exemplifies the clinical utility of therapeutic drug monitoring in oncology [94].

Stoller et al. initially proposed that plasma concentrations exceeding 9 × 10^− 7^ M at 48 h could heighten the risk of myelotoxicity, indicating toxic exposure levels [95]. All consensus guidelines outline folinic acid rescue therapy parameters based on MTX concentration levels. Infusions require to continues until MTX falls between 0.1 and 0.05 µM. However, a 24–36 h delay in measuring plasma MTX is acceptable. While MTX nephrotoxicity correlates with plasma concentration, it poorly predicts other toxicities like myelotoxicity and hepatotoxicity [94]. In acute kidney injury or extremely high MTX blood levels during high-dose MTX therapy, glucapidase (carboxypeptidase G2) is the preferred treatment. Glucapidase efficiently metabolizes ~ 98% of MT from the blood within 30 min, exceeding leucovorin’s capabilities in critical situations. Low-dose MTX (< 50 mg/m^2^) has long been used as a cost-effective disease-modifying agent for rheumatic and autoimmune diseases [96]. Around 40% of patiens do not respond to MTX treatment. Factors like non-compliance, inadequate dosing, and variations in MTX uptake and metabolism contribute to this variability. Cellular MTX uptake, polyglutamation rates, and the activity of the folypolyglutamase synthase enzyme involved in polyglutamation may influence response. MTX polyglutamate levels have been proposed as biomarkers to identify patients at risk of non-response. Research has demonstrated that there is significant variation in the levels of polyglutamate present in patient blood cells even for those receiving the same dose of MTX [97]. De Rotte et al. have proposed a multivariate model including age, gender, folate status, and genotype to correlate disease activity with polyglutamate levels [98]. Moreover, recently, a randomized controlled study showed that daily therapy is as effective as weekly therapy when polyglutamate-3 (PG-3) levels are similar, suggesting that obese patients achieve a lower concentration of MTX-PG3 [99].

For 24 h MTX infusions, TDM is recommended at 24, 48, and 72 h after infusion start until concentrations fall below 0.1–0.2 µmol/L. The 24-h concentration correlates with efficacy and safety, while later concentrations relate primarily to safety. Whereas for rapid infusions under 6 h, the 3–6 h concentration represents peak levels for efficacy and safety evaluation, and later concentrations indicate safety. Generally, MTX levels below 0.1–0.2 µmol/L are considered safe, discontinuing TDM once achieved. However, levels under 0.05 µmol/L may be a stricter safe range for patients with delayed elimination or acute renal dysfunction [100]. In a recent study on improved analysis HPLC-ESI/triple method for mapping the MTX by mass spectrometry. The results obtained from the study suggested that the established HPLC-ESI triple method can accurately and sensitively quantify MTX using only 10 µL of plasma. Moreover, the results suggested that recovery rates for all analytes exceeded 90%, ad matrix effects were minimal. Additionally, the findings suggested that measuring MTX concentration within 24 h of post-administration enhance the effectiveness of monitoring and ensuring optimal therapeutic dosing [101].

#### Busulfan

Busulfan is an alkylating agent used in cancer treatment. It forms reactive ions that crosslink DNA strands, disrupting DNA replication and transcription, ultimately inhibiting cellular proliferation [102]. Busulfan is a widely used chemotherapy agent in high-dose regimens for patients undergoing hematopoietic stem cell transplantation to treat malignant and non-malignant conditions. The standard dosage of busulfan is 1 mg/kg orally four times daily or 3.2 mg/kg intravenously once daily [87].

Busulfan is an effective chemotherapy drug, but optimizing dosing is challenging due to its narrow therapeutic window and low therapeutic index. Inadequate exposure levels can lead to therapeutic failures, while higher exposures can cause short-term and long-term hematological and non-hematological issues. Clinical studies demonstrate a clear relationship between exposure levels, effectiveness, and side effects. Busulfan has a narrow therapeutic window of 900-1200 mMol ⋅ min and a low therapeutic index of < 2, with the threshold for therapeutic response at 900–950 mMol • min and toxic side effects at 1200-1500 mMol ⋅ min [103].

In the early 1980s, shortly after Santos et al. introduced their oral conditioning regimen, a classic case of hepatic veno-occlusive disease was reported. By the late 1980s, it had been proposed that TDM could have a potential role in mitigating such adverse event [104]. Later oral busulfan dosing regimen was modified due to higher exposure correlating with increased adverse events beyond hepatic veno-occlusive disease. These included elevated risks of acute graft-versus-host disease, cytokine release syndrome potentially damaging organs, seizures, and higher treatment-related mortality. While oral administration was the primary factor for veno-occlusive disease, other contributors were concomitant cyclophosphamide, patient susceptibility, and disease characteristics [105]. Grochow and Dix demonstrated that hepatic events are related to drug concentration [104, 106]. The studies revealed that an AUC exceeding 1500 µMol ⋅ min with a steady-state plasma concentration (Css) above 1025 ng/mL were significantly linked to hepatic veno-occlusive disease after 6-h of oral busulfan administration. The evidence confirmed the HVOD threshold to be approximately 1500 µMol ⋅ min for 6 h dosing [107].

Numerous early studies during the oral administration of busulfan documented instances of therapeutic failure. Slattery et al. suggested a concentration cutoff of 1250 µMol ⋅ min and a Css of 925 ng/mL for optimal therapeutic effect in bone marrow transplantation [100]. Subsequently, the results demonstrated that targeting a AUC of > 1350 µMol ⋅ min led to treatment success in patients with chronic myeloid leukemia [108]. In another study Radich et al. also showed similar findings, with an AUC > 1350 µMol ⋅ min correlating with excellent outcomes [109]. More recently, Anderson et al. showed that an AUC value < 900 µMol ⋅ min was associated with higher failure rates in a 6-hourly intravenous busulfan regimen [110].

Children metabolize and eliminate oral busulfan faster than adults, resulting in lower drug exposure. This improved tolerance allows higher doses, but also increases the risk of graft failure after stem cell transplantation due to suboptimal conditioning [111]. Therefore TDM is recommended to adjust the dosing and maintain the area AUC between 900 and 1,500 µMol ⋅ min over 6 h for a 16-dose regimen. Large retrospective studies established this AUC range, corresponding to a cumulative AUC of 144,000-240,000 mMol ⋅ min over 16 doses, for achieving optimal therapeutic effects [112].

#### 5- fluorouracil

5-Fluorouracil, a drug that has been used for over 40 years in management of cancer, inhibits thymidylate synthase, an enzyme crucial for DNA replication. By blocking thymine synthesis, a nucleotide required for DNA replication, 5-FU impedes the rapid proliferation of cancer cells highly reliant on nucleotide synthesis [29]. 5-FU, a chemotherapy drug used since 1978 for colorectal and pancreatic cancers, is metabolized by the enzyme DPD. Variants in the DPD gene, including a point mutation causing a truncated protein, can reduce DPD activity. This DPD deficiency leads to higher 5-FU levels and increased toxicity risk. Considering a patient’s DPD variant status may optimize 5-FU dosing and mitigate adverse effects during cancer treatment [113].

PK variability impacts patient response to treatment. Studies showed that the standard body surface area (BSA)-based dosing method for calculating 5-FU doses is ineffective at achieving optimal plasma concentrations, leading to up to 100-fold variability in patient 5-FU levels and contributing to toxicity and lack of efficacy. Two studies exemplify the limited utility of BSA-based 5-FU dosing, with Gamelin et al. finding no association between BSA and 5-FU clearance in 81 metastatic colorectal cancer patients, and Milano et al. showing a lack of association between BSA and 5-FU clearance in 380 head and neck cancer patients [114, 115].

Clinical studies over the past two decades have consistently shown reduced toxicity and improved outcomes with PK dose management. Direct 5-FU monitoring represents a more tailored dosing method compared to standard body surface area dosing. In a recent study of 208 metastatic colorectal cancer patients, those dosed by body surface area achieved an 18.3% response rate and 16-month survival, while those with PK monitoring had a 33.7% response rate and 22-month survival. The researchers concluded individualized 5-FU dosing based on PK monitoring improved response rates, survival, and reduced adverse effects [116].

#### Osimertinib

Osimertinib have been widely used tyrosine kinase inhibitor for the treatment of non-small cell lung cancer patients with activating EGFR mutations. However, the correlation between dose and efficacy has been debated for several years. Thus, Greibe and co-workers developed and standardized analytical methods for routine analysis, PK and dose-response relationships with stability quantification. The experimental study involve quantification via protein precipitation and separation using Kinetex EVO C18 column. The results demonstrated a validated concentration range between 1.25 and 3000 ng/mL inter-assay precisions and accuracies were ≤ 15%. Moreover, Linearity, dilution integrity, and carry-over were also examined under different conditions, and the analytes were stable for more than 3 years, suggesting that overall method suitability for clinical studies and therapeutic drug monitoring [117].

### mTOR inhibitor

Sirolimus, also known as rapamycin, is an immunosuppressant drug initially developed as an antifungal agent. However, its antiproliferative and immunosuppressive properties were discovered, leading to its use in preventing organ rejection after transplantation. Along with its derivative everolimus, sirolimus belongs to the class of mammalian target of rapamycin (mTOR) inhibitors, which are increasingly employed in cancer treatment [118]. Both sirolimus and everolimus are metabolized by CYP3A4 [119]. Genetic polymorphisms of these enzymes and combinations of drug therapies can impact plasma concentrations of mTOR inhibitors and may result in toxicities or treatment failure [120].

Cattaneo et al. analysed 2,658 sirolimus trough samples from 495 kidney transplant recipients. Researcher found dose-normalized sirolimus trough concentrations were significantly higher in patients treated with cyclosporine and sirolimus compared to those treated with mycophenolate mofetil and sirolimus or steroids and sirolimus [121]. The mean intra- and interpatient variability was 19% an 47%, rspectively. These findings suggest different immunosuppressants can significantly impact sirolimus PK and clinical outcomes. Several studies on everolimus have demonstrated similar potential for interindividual variability and drug-drug interactions as sirolimus [122].

The role of mTOR inhibitors in cancer chemotherapy is complex. Studies show positive responses when added to existing regimens, but no definitive link between drug exposure and desirable clinical outcomes. While drug exposure correlates with side effects, TDM is generally deemed unnecessary for mitigating side effects through early intervention and supportive care. Although TDM is standard for organ transplants, it is rarely used in clinical oncology, except for monitoring immunosuppression in bone marrow transplant patients [123].

### Benefits of therapeutic drug monitoring

#### Identification of drug interactions

Poly-pharmacy is common among cancer patients as new medications are often introduced during anticancer treatment. Potential drug interactions, particularly with CYP3A4 and ABCB1 inhibitors and inducers, have been shown to influence tyrosine kinase inhibitor exposure. Given all the prospective changes and interactions that can occur for a patient over time, it is increasingly important to identify those who require dosage adjustments to achieve appropriate therapeutic drug concentrations while reducing the risk of toxicity [124].

#### Toxicity identification

In many instances, drug toxicity can be clinically diagnosed. However, some adverse effects such as lethargy, loss of appetite, and diarrhoea are non-specific, and determining causality can be difficult to differentiate from malignancy or complications arising from treatments including antibiotics and analgesics. Monitoring drug concentrations may provide useful insights in clarifying whether these symptoms are associated with the targeted agent or other causes [125].

#### Observing dose reduction

Dose reductions of targeted kinase inhibitors due to treatment-related toxicity are common. However, determining the appropriate level of dose decrease to ensure therapeutic efficacy remains challenging. Therapeutic drug monitoring can help track the magnitude of dosage adjustments and prevent an “overshoot” that renders the amount ineffective. It also provides valuable assurance to patients and their oncologists regarding suitable dosing levels.

#### Reducing under dosing

Suboptimal dosing of targeted anticancer therapies can occur inconspicuously, as low doses of these agents may not induce adverse effects that prompt dose escalation. For certain targeted agents, toxicity thresholds are minimal [125]. Whereas a subtherapeutic drug levels correlate with drug resistance and suboptimal outcomes in patients receiving targeted anticancer therapies. While inadequate dosing may be suspected in metastatic cases due to lack of response, such associations are less apparent in the adjuvant setting. TDM could enhance efficacy by facilitating early detection of insufficient exposure, ultimately mitigating undertreatment risk [126].

#### Observing adherence

Historically, oncologists did not regularly examine medication adherence as intravenous drugs are usually administered in a carefully monitored clinical setting. However, with the increasing use of oral targeted cancer therapies, considerations about compliance have become a critical factor. Patient adherence to oral anti-cancer agents varies unpredictably, with adherence rates reported anywhere from 20 to 100% according to studies. Ensuring patients understand dosing instructions and addressing any barriers to treatment can help optimize outcomes for oral regimens. Looking ahead, as oral therapies for cancer see expanded use; adherence will likely remain an important area of focus to improve health outcomes [127].

Non-adherence to prescribed treatment regimens can significantly contribute to variability in a drug’s therapeutic efficacy. Erroneous conclusions from this variability may lead to unnecessary diagnostic testing, hospitalizations, and discontinuing effective treatments. For example, in patients with chronic myeloid leukaemia treated with imatinib for several years, poor adherence has been identified as the main reason for inability to achieve adequate molecular responses. Another commonly overlooked issue is over-adherence to self-administered medications. A “more is better” approach driven by patients or confusion resulting in overdosing has been documented in studies of other diseases and may, in the case of oral cancer therapies, lead to substantially increased toxicity. Therapeutic drug monitoring can be useful for guiding therapeutic decisions when non-adherence, inadequate adherence or excessive adherence is suspected [128].

#### Individualisation in high-risk patient

The introduction of targeted anticancer therapies has enabled a wider group of patients to benefit from their favourable safety profiles and convenient oral dosing options. Specifically, these agents have provided a therapeutic opportunities for older patient cohorts and those with diminished health statuses who may otherwise have difficulty tolerating intensive treatment protocols. The development of targeted agents with improved safety and tolerability has allowed oncologists to help patients who previously had few treatment alternatives, expanding access to potentially life-extending therapies [125].

As individuals aged, their liver’s ability to break down drugs through metabolic enzymes decreases, as does their kidneys’ ability to filter waste from the blood through glomerular filtration. This can result in changes to how drugs are processed in the body over time in elderly patients. Impairment of liver and kidney function is common in cancer patients due to pre-existing organ dysfunction, the spread of cancer to these organs, blockages in the urinary tract, or side effects from cancer treatments. There is limited evidence regarding the effects of liver and kidney impairment on how targeted anti-cancer drugs are handled by the body. However, available data is sometimes inconsistent or even lacking too [129].

## Clinical implementation of therapeutic drug monitoring

Therapeutic drug monitoring in oncology aims to optimize treatment by individualizing drug dosing based on patient-specific factors and drug concentrations. While TDM is well-established in other medical fields, its implementation in oncology faces challenges due to the complexity of cancer treatment and the lack of established therapeutic indices for many anticancer drugs [3]. Recent advancements in sampling strategies, bio-analytics, and dosing decision support present opportunities to overcome these barriers [130]. Moreover, the utilization of TDM has expanded to include pharmacogenetic, demographic, and clinical information, as well as biomarker measurements [1]. Although evidence supporting TDM in oncology is limited, it may play a crucial role in optimizing chemotherapy outcomes, particularly for patients previously ineligible for treatment [3].

Despite its promise, clinical implementation of TDM in oncology remains limited, with most studies focusing on analytical method development rather than clinical outcomes [4]. Economic evaluations have generally found TDM interventions to be cost-effective or cost-saving in cancer care, particularly for drugs like imatinib and 5-fluorouracil [131]. However, challenges persist in conducting comprehensive economic assessments of TDM in oncology, necessitating a structured framework to guide future evaluations [132]. To advance TDM adoption in clinical practice, further research is needed to address evidence gaps, particularly regarding long-term health outcomes and the impact of newer targeted therapies on TDM implementation.

## Limitations in therapeutic drug monitoring

There are several factors currently limiting the clinical utility of TDM for anticancer medications. First, the pharmacology and PK of most anticancer drugs are not fully understood. Second, plasma drug concentration only indirectly estimates the amount of medication in the target tissue because the site of action is often distant from blood vessels and solid tumors may have distinct blood supply patterns. Third, there is a natural lag between measuring a drug’s plasma concentration and assessing its final pharmacodynamic effect, which is often a cure. Improvement in cure rate is frequently the outcome metric used, however accurately assessing the outcome normally requires five years of follow-up [130]. Because of this, research studies are more time-consuming and complex than those for rapidly-acting medications like antibiotics. Fourth, as anti-neoplastic drugs are generally administered together in combinations, it can be challenging to detect concentration-effect relationships. Determining the precise pharmacodynamics of any single agent in light of combination therapy would be quite difficult. Additionally, pharmacodynamic drug toxicity is complicated by combination therapy [133].

## Reported clinical trials and case studies

Clinical trials and case studies on TDM in oncology highlight its potential to optimise cancer treatment by adjusting drug dosages-based on individual PK profiles. Several challenges persist, particularly with oral targeted therapies, where TDM is often not feasible due to high adverse event rates or other pharmacological factors. Despite these challenges, ongoing research and advancements in analytical methods, such as the development of efficient drug measurement systems for tyrosine kinase inhibitors, suggest a promising future for TDM in oncology. Table 4 presents summary recent reports on clinical trials and case studies associated with TDM.

**Table 4:** Summarize recent reports on clinical trials and case studies associated with TDM

Title	Summary	Findings	Limitations	Refs.
TDM of 5-FU	TDM of 5-FU improves clinical outcomes by reducing toxicities.	Dose adjustment of 5-FU-based on PK monitoring to improve efficacy.		[134]
Prospective, multicenter study of 5-FU TDM in metastatic colorectal cancer	TDM of 5-FU improved exposure and reduced toxicities in metastatic colorectal cancer patients.	Personalization of 5-FU dosing using TDM in routine clinical practice resulted in significantly improved 5-FU exposure, with more patients within the target AUC range by the fourth administration. Despite 55% of patients receiving increased 5-FU doses, the incidence of severe toxicities was reduced.	Small sample size of 75 patients across 3 different treatment regimens. Focused on validating the use of TDM rather than evaluating long-term clinical outcomes.	[135]
A retrospective examination of oncology biologics for potential use of therapeutic drug monitoring	Blinatumomab is an anticancer biologic that may benefit from TDM to optimize effectiveness and minimize toxicity.	Five oncology biologics were identified that have established exposure-response relationships for both efficacy and safety: ipilimumab, ziv-aflibercept, necitumumab, brentuximab-vedotin, and blinatumomab. The inter-individual variability in clearance for these 5 biologics ranged from 29–97%. Only 3 of the 5 biologics had a defined maximum tolerated dose.	-	[136]
TDM of monoclonal antibodies	TDM of monoclonal antibodies can guide more effective dosing in individual patients with inflammatory and malignant diseases.	Monoclonal antibodies (mAbs) have considerable interpatient variability in PK, and there are many factors that influence the serum concentration of an mAb. Exposure-response analyses have revealed that patients with low serum trough concentrations of mAbs are at risk of treatment failure.	Establishing the exposure-response relationship and target trough concentration range for each mAb and indication, preferably in prospective clinical trials. Evaluating the optimal timing and scope of mAb-TDM (e.g. at induction, during maintenance, or at loss of response).	[137]
TDM during maintenance therapy with infliximab	Proactive TDM during maintenance infliximab therapy improved disease control, compared to standard therapy in patients with immune-mediated inflammatory diseases.	Proactive TDM during maintenance therapy with infliximab was more effective than standard therapy without TDM in sustaining disease control without disease worsening.	Did not compare proactive TDM to reactive TDM. Due to open-label study possibility of bias is significant.	[138]
Phase-I study of pembrolizumab in advanced solid tumors		The maximum dosage intravenously every two weeks administered was 10 mg/Kg of body weight. Pembrolizumab demonstrated PK properties consistent with those expected for humanized monoclonal antibody therapeutics	-	[139]
TDM of posaconazole in inpatient of acute leukemia	This study quantified plasma posaconazole concentrations (PPCs) in patients receiving prophylactic treatment, assessing factors impacting these levels. Substantial variability in posaconazole bioavailability was observed, with gastrointestinal disorders, food consumption, and concomitant medication administration.	A clinical study of 40 patients on posaconazole prophylaxis obtained 275 plasma PPC measurements. The median PPC was 430 ng/mL. Lower PPC levels correlated with adverse effects like mucositis, nausea, diarrhea, or vomiting. Conversely, higher caloric intake increased PPC.	-	[140]
TDM of Carboplatin with multicentric Phase II trial in germ cell tumours patient	A phase II clinical trial explored high-dose chemotherapy with the TI-CE regimen for relapsed, advanced germ cell tumors. TDM of carboplatin enabled optimizing the area AUC for this agent. Individualized tracking of carboplatin plasma concentrations allowed precise control over the target AUC, preventing under- or over-exposure	The study found notable full and positive outcomes among these high-risk patients. Tailored monitoring of carboplatin levels in the blood allowed for more accurate regulation of the target AUC.	-	[141]
TDM of Pazopanib	This study evaluated the feasibility of utilizing PK-guided individualized dosing to mitigate interpatient variability in pazopanib exposure, measured by the area under the plasma concentration-time curve (AUC_0 − 24 h_). Notably, the intra-patient variability in pazopanib exposure observed in this study was relatively high, compared to the interpatient variability	The implementation of PK-guided dosing did not diminish the variability in pazopanib exposure among patients. The intra-patient variability in pazopanib exposure was substantial, compared to the interpatient variability. To effectively reduce interpatient variability through PK -guided dosing, it is necessary first to understand the causes of intra-patient variability and implement measures to control it		[142]

## Emerging trends and future directions

Artificial intelligence and machine learning are rapidly advancing in oncology, offering promising applications across the cancer care continuum. These technologies are being utilized for risk assessment, automated image analysis, lesion detection, and treatment response prediction [143]. Moreover, this approach integrates vast datasets, including genomic, proteomic, and clinical information, to predict patient responses to cancer treatments. Recent advancements include whole blood multi-cancer detection, virtual biopsies, and advanced clinical decision support systems [144]. AI is also optimizing anticancer drug research, although limitations still exist [145]. AI algorithms can identify novel biomarkers and therapeutic targets, enhancing precision medicine by trailering treatment strategies to individual patients. Platforms like CURATE.AI use AI to optimize drug dosing by developing personalized “digital avatars” for patients, ensuring safer and more effective treatment. Quantitatively, AI-driven models have shown high accuracy in predicting patient’s outcomes, such as survival times, and in identifying subsets of patients likely to benefits from specific therapies. Furthermore, in clinical trials, AI has shown potential in predicting acute care visits, short-term mortality, and pathologic extra nodal extension [146]. Despite these advancements, challenges remain, including data transparency, interpretability, and potential biases against underrepresented populations. Future directions involve increasing model complexity using multimodal data elements and developing living databases for highly personalized treatment approaches.

Next-generation sequencing and omics technologies have revolutionized oncology research and clinical practice. These advanced techniques enable comprehensive profiling of tumors at genomic, transcriptomic, and proteomic levels, providing insights into cancer etiopathogenesis and driving personalized medicine approaches [147]. Transcriptomics, in particular, has emerged as a crucial tool for identifying cancer biomarkers, gene signatures, and molecular targets for therapy [148]. The integration of multi-omics data, including spatial genomics, transcriptomics, and proteomics, offers high-resolution tumor profiling that can inform precision treatment strategies. Next-generation sequencing applications in clinical oncology range from discovering novel mutations to guiding personalized therapies, potentially reducing medical expenses by shifting towards preventive and predictive approaches [149]. While these technologies have significantly advanced our understanding of cancer biology and treatment options, challenges remain in translating high-resolution tumor profiling into clinical practice.

TDM in oncology has the potential to improve treatment outcomes by personalizing drug dosages based on individual patient blood levels [7]. However, widespread implementation faces challenges such as logistical complexity, lack of clinical laboratories, and insufficient reimbursement [150]. The main obstacle is the scarcity of studies demonstrating improved clinical outcomes with TDM-guided dosing. Recent advancements in sampling strategies, bio-analytics, and dosing decision support offer opportunities to address these limitations. Point-of-care TDM, combining sensitive analytical methods with model-informed precision dosing platforms, could enable real-time dose adjustments for cytotoxic chemotherapies [151]. Liquid chromatography-mass spectrometry-based TDM shows promise for precision medicine applications. Overcoming these challenges could lead to improved patient outcomes and reduced mortality rates in oncology.

## Conclusion

TDM plays an increasingly vital role in modern oncology and offers a critical tool for optimizing cancer treatment by ensuring patients receive the right dose of medication. Given the complexity of cancer pharmacotherapy, narrow therapeutic windows, and significant inter-individual variability in PK, TDM is indispensable for enhancing both the safety and effectiveness of cancer treatments. Moreover, through analytical techniques and quantifications, TDM helps address the significant interpatient variability in drug exposure, which is particularly important for drugs with narrow therapeutic windows. Current methods like pharmacogenetic testing, immunoassays, and LC-MS/MS have shown promise, but challenges such as the need for robust clinical evidence, standardized practices, and seamless integration with personalized medicine remain. Although, TDM endures challenges, ongoing AI-based predictive modelling and the expansion of TDM guidelines to newer targeted treatments suggest a promising future for personalized oncology care. As research continues to bridge the gaps in exposure-response relationships and population PK, TDM is poised to play an increasingly vital role in improving patient outcomes in oncology.

## Data Availability

No datasets were generated or analysed during the current study.

## Abbreviations

TDM: Therapeutic drug monitoring
LC: Liquid chromatography
UV: Ultra-violet
MS: Mass spectroscopy
TMS: Tandem mass spectrometry
LC-MS: Liquid Chromatography-Mass Spectroscopy
HPLC: High-Performance Liquid Chromatography
AUC: Area Under Curve
PK: Pharmacokinetic
AAG: Alpha 1-acid glycoprotein
CYP450: Cytochrome P450
ABC: ATP-Binding cassettes
MTX: Methotrexate
HPLC-UV: High Performance Liquid Chromatography-Ultraviolet
FLD: Fluorescence detection
DDE: 1,1-(o, p′-dichlorodiphenyl)-2,2 dichloroethene
DDA: 1,1-(o, p′-dichlorodiphenyl) acetic acid
5-FU: 5-fluorouracil
SERS: Surface Enhanced Raman Spectroscopy
6-MP: 6-mercaptopurine
TLC: Thin Layer Chromatography
LLE: Liquid-liquid extractions
SPE: Solid phase Extraction
ACN: Acetonitrile
DPD: dihydro pyrimidine dehydrogenase
HSCT: hematopoietic stem cell transplantation
NGS: Next-generation sequencing
PET: Positron Emission Tomography
MRI: Magnetic Resonance Imaging
DNA: Deoxyribonucleic acid
RNA: Ribonucleic acid
HDMTX: High-dose Methotrexate
PG-3: Polyglutamate-3
mAbs: Monoclonal antibodies
PPCs: Plasma posaconazole concentrations

## References

[1] Wilkinson DS. Therapeutic drug monitoring in oncology. Ther Drug Monit. 2019;41:551–2. 31318845 10.1097/FTD.0000000000000679

[2] Bardin C, Veal G, Paci A, Chatelut E, Astier A, Levêque D, et al. Therapeutic drug monitoring in cancer– Are we missing a trick? Eur J Cancer. 2014;50:2005–9. 24878063 10.1016/j.ejca.2014.04.013

[3] Saleem M, Dimeski G, Kirkpatrick CM, Taylor PJ, Martin JH. Target concentration intervention in oncology. Ther Drug Monit. 2012;34:257–65. 22585183 10.1097/FTD.0b013e3182557342

[4] Stojanova J, Carland JE, Murnion B, Seah V, Siderov J, Lemaitre F. Therapeutic drug monitoring in oncology - What’s out there: A bibliometric evaluation on the topic. Front Oncol. 2022;12. 10.3389/fonc.2022.959741 PMC9685987 36439413

[5] Clarke WA, Chatelut E, Fotoohi AK, Larson RA, Martin JH, Mathijssen RHJ, et al. Therapeutic drug monitoring in oncology: international association of therapeutic drug monitoring and clinical toxicology consensus guidelines for Imatinib therapy. Eur J Cancer. 2021;157:428–40. 34597977 10.1016/j.ejca.2021.08.033

[6] Knezevic CE, Clarke W. Cancer chemotherapy: the case for therapeutic drug monitoring. Ther Drug Monit. 2020;42:6–19. 31568180 10.1097/FTD.0000000000000701

[7] Lyashchenko AK, Cremers S. On precision dosing of oral small molecule drugs in oncology. Br J Clin Pharmacol. 2021;87:263–70. 32621551 10.1111/bcp.14454 PMC8938983

[8] Mishra AP, Kumar R, Harilal S, Nigam M, Datta D, Singh S, et al. Demystifying the management of cancer through smart nano-biomedicine via regulation of reactive oxygen species. Naunyn-Schmiedeberg’s Archives Pharmacol 2024. 2024;398(1):398:497–532. 10.1007/s00210-024-03469-x 39480523

[9] Morgan LR, Weatherall TJ. Pharmacology and drug distribution. Int J Radiat Oncol Biol Phys. 1979;5:1205–12. 575116 10.1016/0360-3016(79)90640-0

[10] Lewis LD. Cancer pharmacotherapy: 21st century ‘magic bullets’ and changing paradigms. Br J Clin Pharmacol. 2006;62:1–4. 16842373 10.1111/j.1365-2125.2006.02721.x PMC1885080

[11] Urruticoechea A, Alemany R, Balart J, Villanueva A, Vinals F, Capella G. Recent advances in cancer therapy: an overview. Curr Pharm Des. 2010;16:3–10. 20214614 10.2174/138161210789941847

[12] Bray F, Laversanne M, Weiderpass E, Soerjomataram I. The ever-increasing importance of cancer as a leading cause of premature death worldwide. Cancer. 2021;127:3029–30. 34086348 10.1002/cncr.33587

[13] Chen S, Cao Z, Prettner K, Kuhn M, Yang J, Jiao L, et al. Estimates and projections of the global economic cost of 29 cancers in 204 countries and territories from 2020 to 2050. JAMA Oncol. 2023;9:465–72. 36821107 10.1001/jamaoncol.2022.7826 PMC9951101

[14] Qazi AS. Introduction and Overview of Cancer Therapeutics. 2023. pp. 1–13. 10.1007/978-3-031-27156-4_1 37306901

[15] Asif QUA. Cancer Pharmacology advances: fundamentals, combination, imagery, shipment and therapeutic applications. Indian J Pure Appl Biosci. 2023;11:1–9.

[16] Jimenez PC, Wilke DV, Branco PC, Bauermeister A, Rezende-Teixeira P, Gaudêncio SP, et al. Enriching cancer Pharmacology with drugs of marine origin. Br J Pharmacol. 2020;177:3–27. 31621891 10.1111/bph.14876 PMC6976878

[17] Blebea NM, Bucur LA. Pharmacotherapeutic options in neoplastic diseases– part II. Farmacist Ro. 2021;2:18.

[18] Makimbetov EK, Salikhar RI, Tumanbaev AM, Toktanalieva AN, Kerimov AD. Cancer epidemiology in the world. Современные Проблемы Науки И Образования (Modern Probl Sci Education). 2020;–2 2020:133–133.

[19] Ferlay J, Shin H, Bray F, Forman D, Mathers C, Parkin DM. Estimates of worldwide burden of cancer in 2008: Globocan 2008. Int J Cancer. 2010;127:2893–917. 21351269 10.1002/ijc.25516

[20] Hesketh R. Lessons from epidemiology. Introduction to cancer biology. Cambridge University Press; 2012. pp. 1–19.

[21] Mattiuzzi C, Lippi G. Current cancer epidemiology. J Epidemiol Glob Health. 2019;9:217. 31854162 10.2991/jegh.k.191008.001 PMC7310786

[22] Sung H, Ferlay J, Siegel RL, Laversanne M, Soerjomataram I, Jemal A, et al. Global cancer statistics 2020: Globocan estimates of incidence and mortality worldwide for 36 cancers in 185 countries. CA Cancer J Clin. 2021;71:209–49. 33538338 10.3322/caac.21660

[23] Nematullah M, Hasmatullah, Agnihotri A, Kumar S, Husain A, Rahman MA. Evaluation of therapeutics’ drug monitoring during cancer chemotherapy: A review. Intell Pharm. 2023;1:157–61.

[24] van Brummelen EMJ, Huitema ADR, van Werkhoven E, Beijnen JH, Schellens JHM. The performance of model-based versus rule-based phase I clinical trials in oncology. J Pharmacokinet Pharmacodyn. 2016;43:235–42. 26960536 10.1007/s10928-016-9466-0

[25] Dasgupta A. Introduction to therapeutic drug monitoring. Handbook of drug monitoring methods. Totowa: Humana; 2008. pp. 1–39.

[26] Kang J-S, Lee M-H. Overview of therapeutic drug monitoring. Korean J Intern Med. 2009;24:1. 19270474 10.3904/kjim.2009.24.1.1 PMC2687654

[27] Noda S, Morita S, Terada T. Dose individualization of oral Multi-Kinase inhibitors for the implementation of therapeutic drug monitoring. Biol Pharm Bull. 2022;45:b21–01098. 10.1248/bpb.b21-01098 35786588

[28] Burtis CA, Ashwood ER, Bruns DE. Tietz fundamentals of clinical chemistry. Saunders; 2008.

[29] Bach DM, Straseski JA, Clarke W. Therapeutic drug monitoring in cancer chemotherapy. Bioanalysis. 2010;2:863–79. 21083218 10.4155/bio.10.48

[30] Sawicki E, Schellens JHM, Beijnen JH, Nuijen B. Inventory of oral anticancer agents: pharmaceutical formulation aspects with focus on the solid dispersion technique. Cancer Treat Rev. 2016;50:247–63. 27776286 10.1016/j.ctrv.2016.09.012

[31] Undevia SD, Gomez-Abuin G, Ratain MJ. Pharmacokinetic variability of anticancer agents. Nat Rev Cancer. 2005;5:447–58. 15928675 10.1038/nrc1629

[32] Slaviero KA, Clarke SJ, Rivory LP. Inflammatory response: an unrecognised source of variability in the pharmacokinetics and pharmacodynamics of cancer chemotherapy. Lancet Oncol. 2003;4:224–32. 12681266 10.1016/s1470-2045(03)01034-9

[33] Innocenti F, Undevia SD, Iyer L, Xian Chen P, Das S, Kocherginsky M, et al. Genetic variants in the *UDP-glucuronosyltransferase 1A1* gene predict the risk of severe neutropenia of Irinotecan. J Clin Oncol. 2004;22:1382–8. 15007088 10.1200/JCO.2004.07.173

[34] Beutler E, Gelbart T, Demina A. Racial variability in the UDP-glucuronosyltransferase 1 (UGT1A1) promoter: A balanced polymorphism for regulation of bilirubin metabolism? Proc Natl Acad Sci. 1998;95:8170–4. 9653159 10.1073/pnas.95.14.8170 PMC20948

[35] Nozawa T, Minami H, Sugiura S, Tsuji A, Tamai I. Role of organic anion transporter oatp1b1 (oatp-c) in hepatic uptake of Irinotecan and its active metabolite, 7-ethyl-10-hydroxycamptothecin: in vitro evidence and effect of single nucleotide polymorphisms. Drug Metab Dispos. 2005;33:434–9. 15608127 10.1124/dmd.104.001909

[36] Lippens RJ. Methotrexate. I. Pharmacology and pharmacokinetics. Am J Pediatr Hematol Oncol. 1984;6:379–95. 6398629

[37] Vestal RE. Aging and Pharmacology. Cancer. 1997;80:1302–10. 9317183 10.1002/(sici)1097-0142(19971001)80:7<1302::aid-cncr16>3.0.co;2-b

[38] Sotaniemi EA, Arranto AJ, Pelkonen O, Pasanen M. Age and cytochrome P450-linked drug metabolism in humans: an analysis of 226 subjects with equal histopathologic conditions. Clin Pharmacol Ther. 1997;61:331–9. 9091249 10.1016/S0009-9236(97)90166-1

[39] Anderson S, Brenner BM. Effects of aging on the renal glomerulus. Am J Med. 1986;80:435–42. 3513560 10.1016/0002-9343(86)90718-7

[40] Epstein FH, Brenner BM, Meyer TW, Hostetter TH. Dietary protein intake and the progressive nature of kidney disease. N Engl J Med. 1982;307:652–9. 7050706 10.1056/NEJM198209093071104

[41] Jin Y, Desta Z, Stearns V, Ward B, Ho H, Lee K-H, et al. CYP2D6 genotype, antidepressant use, and Tamoxifen metabolism during adjuvant breast cancer treatment. JNCI J Natl Cancer Inst. 2005;97:30–9. 15632378 10.1093/jnci/dji005

[42] Tuzimski T, Petruczynik A. Review of chromatographic methods coupled with modern detection techniques applied in the therapeutic drugs monitoring (TDM). Molecules. 2020;25:4026. 32899296 10.3390/molecules25174026 PMC7504794

[43] Adaway JE, Keevil BG. Therapeutic drug monitoring and LC–MS/MS. J Chromatogr B. 2012;883–884:33–49. 10.1016/j.jchromb.2011.09.041 21992751

[44] Dasgupta A, Datta P. Analytical techniques for measuring concentrations of therapeutic drugs in biological fluids. Handbook of drug monitoring methods. Totowa, NJ: Humana; 2008. pp. 67–86.

[45] Jain S, Jadav T, Sahu AK, Kalia K, Sengupta P. An exploration of advancement in analytical methodology for quantification of anticancer drugs in biometrics. Japan Soc Anal Chem. 2019;35:719–32. 10.2116/analsci.19R002 30905906

[46] Abumiya M, Miura M, Takahashi N. Therapeutic drug monitoring of Ponatinib using a simple high-performance liquid chromatography method in Japanese patients. Leuk Res. 2018;64:42–5. 29175427 10.1016/j.leukres.2017.11.012

[47] Treder N, Maliszewska O, Olędzka I, Kowalski P, Miękus N, Bączek T, et al. Development and validation of a high-performance liquid chromatographic method with a fluorescence detector for the analysis of epirubicin in human urine and plasma, and its application in drug monitoring. J Chromatogr B. 2020;1136:121910. 10.1016/j.jchromb.2019.121910 31830661

[48] Zhang M, Zhang Y, Ren S, Zhang Z, Wang Y, Song R. Optimization of a precolumn OPA derivatization HPLC assay for monitoring of l-Asparagine depletion in serum during l-Asparaginase therapy. J Chromatogr Sci. 2018;56:794–801. 29878070 10.1093/chromsci/bmy053

[49] Koller D, Vaitsekhovich V, Mba C, Steegmann JL, Zubiaur P, Abad-Santos F, et al. Effective quantification of 11 tyrosine kinase inhibitors and caffeine in human plasma by validated LC-MS/MS method with potent phospholipids clean-up procedure. Application to therapeutic drug monitoring. Talanta. 2020;208:120450. 31816725 10.1016/j.talanta.2019.120450

[50] Wu D, Wang Y, Sun Y, Ouyang N, Qian J. A simple, rapid and reliable liquid chromatography–mass spectrometry method for determination of methotrexate in human plasma and its application to therapeutic drug monitoring. Biomed Chromatogr. 2015;29:1197–202. 25641007 10.1002/bmc.3408

[51] Marangon E, Buzzo M, Posocco B, Gagno S, Zanchetta M, Iacuzzi V, et al. A new high-performance liquid chromatography–tandem mass spectrometry method for the determination of Sunitinib and N-desethyl Sunitinib in human plasma: Light-induced isomerism overtaking towards therapeutic drug monitoring in clinical routine. J Pharm Biomed Anal. 2020;179:112949. 31784210 10.1016/j.jpba.2019.112949

[52] Birch M, Morgan PE, Handley S, Ho A, Ireland R, Flanagan RJ. Simple methodology for the therapeutic drug monitoring of the tyrosine kinase inhibitors dasatinib and Imatinib. Biomed Chromatogr. 2013;27:335–42. 22886846 10.1002/bmc.2796

[53] Feliu C, Cazaubon Y, Guillemin H, Vautier D, Oget O, Millart H et al. Therapeutic drug monitoring of mitotane: analytical assay and patient follow-up. Biomed Chromatogr. 2017;31. 10.1002/bmc.3993 28432798

[54] Cusato J, De Francia S, Allegra S, Carrella S, Pirro E, Piccione FM, et al. Circannual variation of mitotane and its metabolites plasma levels in patients with adrenocortical carcinoma†‡. J Pharm Pharmacol. 2017;69:1524–30. 28809444 10.1111/jphp.12798

[55] Büchel B, Rhyn P, Schürch S, Bühr C, Amstutz U, Largiadèr R. LC-MS/MS method for simultaneous analysis of uracil, 5,6‐dihydrouracil, 5‐fluorouracil and 5‐fluoro‐5,6‐dihydrouracil in human plasma for therapeutic drug monitoring and toxicity prediction in cancer patients. Biomed Chromatogr. 2013;27:7–16. 22454320 10.1002/bmc.2741

[56] Antunes MV, Rosa DD, Viana dos T, Andreolla S, Fontanive H, Linden TO. Sensitive HPLC–PDA determination of Tamoxifen and its metabolites N-desmethyltamoxifen, 4-hydroxytamoxifen and Endoxifen in human plasma. J Pharm Biomed Anal. 2013;76:13–20. 23291438 10.1016/j.jpba.2012.12.005

[57] Lankheet NAG, Hillebrand MJX, Rosing H, Schellens JHM, Beijnen JH, Huitema ADR. Method development and validation for the quantification of dasatinib, erlotinib, gefitinib, imatinib, lapatinib, nilotinib, Sorafenib and Sunitinib in human plasma by liquid chromatography coupled with tandem mass spectrometry. Biomed Chromatogr. 2013;27:466–76. 22987603 10.1002/bmc.2814

[58] Pirro E, De Francia S, De Martino F, Racca S, Di Carlo F, Fava C, et al. A new HPLC-UV validated method for therapeutic drug monitoring of tyrosine kinase inhibitors in leukemic patients. J Chromatogr Sci. 2011;49:753–7. 22080802 10.1093/chrsci/49.10.753

[59] D’Avolio A, Simiele M, De Francia S, Ariaudo A, Baietto L, Cusato J, et al. HPLC–MS method for the simultaneous quantification of the antileukemia drugs imatinib, dasatinib and nilotinib in human peripheral blood mononuclear cell (PBMC). J Pharm Biomed Anal. 2012;59:109–16. 22036594 10.1016/j.jpba.2011.10.003

[60] Yasu T, Momo K, Yasui H, Kuroda S. Simple determination of plasma ibrutinib concentration using high-performance liquid chromatography. Biomed Chromatogr. 2019;33. 10.1002/bmc.4435 30421802

[61] Liao C, Chang S, Hu S, Tang Z, Fu G. Rapid and sensitive liquid chromatography–tandem mass spectrometry method for determination of 1-β-d-arabinofuranosyluracil in human plasma and application to therapeutic drug monitoring in patient with leukemia. J Pharm Biomed Anal. 2013;85:118–22. 23933564 10.1016/j.jpba.2013.07.015

[62] Legrand T, Rakotoson M-G, Galactéros F, Bartolucci P, Hulin A. Determination of hydroxyurea in human plasma by HPLC-UV using derivatization with xanthydrol. J Chromatogr B. 2017;1064:85–91. 10.1016/j.jchromb.2017.09.008 28915422

[63] Mills JR, Cornec D, Dasari S, Ladwig PM, Hummel AM, Cheu M, et al. Using mass spectrometry to quantify rituximab and perform individualized Immunoglobulin phenotyping in ANCA-Associated vasculitis. Anal Chem. 2016;88:6317–25. 27228216 10.1021/acs.analchem.6b00544

[64] Begas E, Papandreou C, Tsakalof A, Daliani D, Papatsibas G, Asprodini E. Simple and reliable HPLC method for the monitoring of methotrexate in osteosarcoma patients. J Chromatogr Sci. 2014;52:590–5. 23800772 10.1093/chromsci/bmt081

[65] Montemurro M, De Zan MM, Robles JC. Optimized high performance liquid chromatography–ultraviolet detection method using core-shell particles for the therapeutic monitoring of methotrexate. J Pharm Anal. 2016;6:103–11. 29403969 10.1016/j.jpha.2015.12.001 PMC5762447

[66] Jaworska A, Fornasaro S, Sergo V, Bonifacio A. Potential of surface enhanced Raman spectroscopy (SERS) in therapeutic drug monitoring (TDM). Crit Rev Biosens (Basel). 2016;6:47. 10.3390/bios6030047 PMC5039666 27657146

[67] Schlücker S. SERS microscopy: nanoparticle probes and biomedical applications. ChemPhysChem. 2009;10:1344–54. 19565576 10.1002/cphc.200900119

[68] Haynes CL, McFarland AD, Van Duyne RP. Surface-Enhanced Raman Spectroscopy. Anal Chem. 2005;77:338 A-346 A.

[69] Farquharson S, Gift A, Shende C, Inscore F, Ordway B, Farquharson C, et al. Surface-enhanced Raman spectral measurements of 5-Fluorouracil in saliva. Molecules. 2008;13:2608–27. 18946423 10.3390/molecules13102608 PMC6245365

[70] Fornasaro S, Marta SD, Rabusin M, Bonifacio A, Sergo V. Toward SERS-based point-of-care approaches for therapeutic drug monitoring: the case of methotrexate. Faraday Discuss. 2016;187:485–99. 27055173 10.1039/c5fd00173k

[71] Yang L, Chen Y, Li H, Luo L, Zhao Y, Zhang H, et al. Application of silver nanoparticles decorated with β-cyclodextrin in determination of 6-mercaptopurine by surface-enhanced Raman spectroscopy. Anal Methods. 2015;7:6520–7.

[72] Fu WL, Zhen SJ, Huang CZ. Controllable Preparation of graphene oxide/metal nanoparticle hybrids as surface-enhanced Raman scattering substrates for 6-mercaptopurine detection. RSC Adv. 2014;4:16327–32.

[73] Farquharson S, Gift AD, Shende C, Maksymiuk P, Inscore FE, Murran J. Detection of 5-fluorouracil in saliva using surface-enhanced Raman spectroscopy. Vib Spectrosc. 2005;38:79–84.

[74] Cottat M, Lidgi-Guigui N, Hamouda F, Bartenlian B, Venkataraman D, Marks RS, et al. Highly sensitive detection of Paclitaxel by surface-enhanced Raman scattering. J Opt. 2015;17:114019.

[75] Yan T, Gu H, Yuan X, Wu J, Wei H. Surface-enhanced Raman spectroscopy study of the interaction of antitumoral drug Paclitaxel with human serum albumin. In: Luo Q, Wang L V., Tuchin V V., editors. 2008. p. 72800 N.

[76] Yuen C, Zheng W, Huang Z. Low-level detection of anti-cancer drug in blood plasma using microwave-treated gold-polystyrene beads as surface-enhanced Raman scattering substrates. Biosens Bioelectron. 2010;26:580–4. 20709521 10.1016/j.bios.2010.07.030

[77] Rath S, Sahu A, Gota V, Martínez-Torres PG, Pichardo-Molina JL, Murali Krishna C. Raman spectroscopy for detection of Imatinib in plasma: A proof of concept. J Innov Opt Health Sci. 2015;08:1550019.

[78] Lorén A, Eliasson C, Josefson M, Murty KVGK, Käll M, Abrahamsson J, et al. Feasibility of quantitative determination of doxorubicin with surface-enhanced Raman spectroscopy. J Raman Spectrosc. 2001;32:971–4.

[79] McLaughlin C, MacMillan D, McCardle C, Smith WE. Quantitative analysis of Mitoxantrone by Surface-Enhanced resonance Raman scattering. Anal Chem. 2002;74:3160–7. 12141678 10.1021/ac010067k

[80] Wu H-Y, Cunningham BT. Point-of-care detection and real-time monitoring of intravenously delivered drugs via tubing with an integrated SERS sensor. Nanoscale. 2014;6:5162–71. 24699532 10.1039/c4nr00027g

[81] Hidi IJ, Mühlig A, Jahn M, Liebold F, Cialla D, Weber K, et al. LOC-SERS: towards point-of-care diagnostic of methotrexate. Anal Methods. 2014;6:3943–7.

[82] Yang J, Tan X, Shih W-C, Cheng MM-C. A sandwich substrate for ultrasensitive and label-free SERS spectroscopic detection of folic acid / methotrexate. Biomed Microdevices. 2014;16:673–9. 24850231 10.1007/s10544-014-9871-3

[83] Li Y-T, Qu L-L, Li D-W, Song Q-X, Fathi F, Long Y-T. Rapid and sensitive in-situ detection of Polar antibiotics in water using a disposable Ag–graphene sensor based on electrophoretic preconcentration and surface-enhanced Raman spectroscopy. Biosens Bioelectron. 2013;43:94–100. 23287654 10.1016/j.bios.2012.12.005

[84] Vicario A, Sergo V, Toffoli G, Bonifacio A. Surface-enhanced Raman spectroscopy of the anti-cancer drug Irinotecan in presence of human serum albumin. Colloids Surf B Biointerfaces. 2015;127:41–6. 25645751 10.1016/j.colsurfb.2015.01.023

[85] Zhao SS, Bukar N, Toulouse JL, Pelechacz D, Robitaille R, Pelletier JN, et al. Miniature multi-channel SPR instrument for methotrexate monitoring in clinical samples. Biosens Bioelectron. 2015;64:664–70. 25441416 10.1016/j.bios.2014.09.082

[86] Blaszykowski C, Sheikh S, Thompson M. Surface chemistry to minimize fouling from blood-based fluids. Chem Soc Rev. 2012;41:5599. 22772072 10.1039/c2cs35170f

[87] Salman B, Al-Khabori M. Applications and challenges in therapeutic drug monitoring of cancer treatment: A review. J Oncol Pharm Pract. 2021;27:693–701. 33302823 10.1177/1078155220979048

[88] Bertholee D, Maring JG, van Kuilenburg ABP. Genotypes affecting the pharmacokinetics of anticancer drugs. Clin Pharmacokinet. 2017;56:317–37. 27641154 10.1007/s40262-016-0450-z PMC5340837

[89] Evans WE, Relling MV, Rodman JH, Crom WR, Boyett JM, Pui C-H. Conventional compared with individualized chemotherapy for childhood acute lymphoblastic leukemia. N Engl J Med. 1998;338:499–505. 9468466 10.1056/NEJM199802193380803

[90] Schmiegelow K. Advances in individual prediction of methotrexate toxicity: a review. Br J Haematol. 2009;146:489–503. 19538530 10.1111/j.1365-2141.2009.07765.x

[91] Wiczer T, Dotson E, Tuten A, Phillips G, Maddocks K. Evaluation of incidence and risk factors for high-dose methotrexate-induced nephrotoxicity. J Oncol Pharm Pract. 2016;22:430–6. 26152702 10.1177/1078155215594417

[92] Levêque D, Becker G, Toussaint E, Fornecker L-M, Paillard C. Clinical pharmacokinetics of methotrexate in oncology. Int J Pharmacokinet. 2017;2:137–47.

[93] Joerger M, Ferreri AJM, Krähenbühl S, Schellens JHM, Cerny T, Zucca E, et al. Dosing algorithm to target a predefined AUC in patients with primary central nervous system lymphoma receiving high dose methotrexate. Br J Clin Pharmacol. 2012;73:240–7. 21838788 10.1111/j.1365-2125.2011.04084.x PMC3269583

[94] Howard SC, McCormick J, Pui C-H, Buddington RK, Harvey RD. Preventing and managing toxicities of High-Dose methotrexate. Oncologist. 2016;21:1471–82. 27496039 10.1634/theoncologist.2015-0164 PMC5153332

[95] Stoller RG, Hande KR, Jacobs SA, Rosenberg SA, Chabner BA. Use of plasma pharmacokinetics to predict and prevent methotrexate toxicity. N Engl J Med. 1977;297:630–4. 302412 10.1056/NEJM197709222971203

[96] Saag KG, Teng GG, Patkar NM, Anuntiyo J, Finney C, Curtis JR, et al. American college of rheumatology 2008 recommendations for the use of nonbiologic and biologic disease-modifying antirheumatic drugs in rheumatoid arthritis. Arthritis Care Res (Hoboken). 2008;59:762–84. 10.1002/art.23721 18512708

[97] Muller IB, Hebing RF, Jansen G, Nurmohamed MT, Lems WF, Peters GJ, et al. Personalized medicine in rheumatoid arthritis: methotrexate polyglutamylation revisited. Expert Rev Precis Med Drug Dev. 2018;3:331–4.

[98] de Rotte MCFJ, Pluijm SMF, de Jong PHP, Bulatović Ćalasan M, Wulffraat NM, Weel AEAM, et al. Development and validation of a prognostic multivariable model to predict insufficient clinical response to methotrexate in rheumatoid arthritis. PLoS ONE. 2018;13:e0208534. 30532219 10.1371/journal.pone.0208534 PMC6287811

[99] Rodríguez-Báez AS, Huerta‐García AP, Medellín‐Garibay SE, Rodríguez‐Pinal CJ, Martínez‐Martínez MU, Herrera‐Van Oostdam D, et al. Disease activity and therapeutic drug monitoring of polyglutamates of methotrexate after daily or weekly administration of low‐dose methotrexate in patients recently diagnosed with rheumatoid arthritis. Basic Clin Pharmacol Toxicol. 2022;130:644–54. 35365958 10.1111/bcpt.13728

[100] Smita P, Narayan PA, Gaurav JK. P. Therapeutic drug monitoring for cytotoxic anticancer drugs: principles and evidence-based practices. Front Oncol. 2022;12. 10.3389/fonc.2022.1015200 PMC9773989 36568145

[101] Jia H, Li R, Li Y, Lu F, Ma L, Xu X. Improved analysis HPLC-ESI/triple method for mapping the methotrexate by mass spectrometry. J Chromatogr B. 2025;1255:124529. 10.1016/j.jchromb.2025.124529 39987857

[102] Bleyzac N, Souillet G, Magron P, Janoly A, Martin P, Bertrand Y, et al. Improved clinical outcome of paediatric bone marrow recipients using a test dose and bayesian Pharmacokinetic individualization of Busulfan dosage regimens. Bone Marrow Transpl. 2001;28:743–51. 10.1038/sj.bmt.1703207 11781625

[103] Geddes M, Kangarloo SB, Naveed F, Quinlan D, Chaudhry MA, Stewart D, et al. High Busulfan exposure is associated with worse outcomes in a daily i.v. Busulfan and fludarabine allogeneic transplant regimen. Biol Blood Marrow Transplant. 2008;14:220–8. 18215782 10.1016/j.bbmt.2007.10.028

[104] Dix SP, Wingard JR, Mullins RE, Jerkunica I, Davidson TG, Gilmore CE, et al. Association of Busulfan area under the curve with veno-occlusive disease following BMT. Bone Marrow Transpl. 1996;17:225–30. 8640171

[105] Ciurea SO, Andersson BS. Busulfan in hematopoietic stem cell transplantation. Biol Blood Marrow Transplant. 2009;15:523–36. 19361744 10.1016/j.bbmt.2008.12.489 PMC4261695

[106] Grochow LB. Busulfan disposition: the role of therapeutic monitoring in bone marrow transplantation induction regimens. Semin Oncol. 1993;20(4 Suppl 4):18–25. quiz 26. 8342071

[107] Slattery JT, Sanders JE, Buckner CD, Schaffer RL, Lambert KW, Langer FP, et al. Graft-rejection and toxicity following bone marrow transplantation in relation to Busulfan pharmacokinetics. Bone Marrow Transpl. 1995;16:31–42. 7581127

[108] Slattery JT, Clift RA, Buckner CD, Radich J, Storer B, Bensinger WI, et al. Marrow transplantation for chronic myeloid leukemia: the influence of plasma Busulfan levels on the outcome of transplantation. Blood. 1997;89:3055–60. 9108427

[109] Radich JP, Gooley T, Bensinger W, Chauncey T, Clift R, Flowers M, et al. HLA-matched related hematopoietic cell transplantation for chronic-phase CML using a targeted Busulfan and cyclophosphamide preparative regimen. Blood. 2003;102:31–5. 12595317 10.1182/blood-2002-08-2619

[110] Andersson BS, Thall PF, Madden T, Couriel D, Wang X, Tran HT, et al. Busulfan systemic exposure relative to regimen-related toxicity and acute graft-versus-host disease: defining a therapeutic window for i.v. BuCy2 in chronic myelogenous leukemia. Biol Blood Marrow Transplant. 2002;8:477–85. 12374452 10.1053/bbmt.2002.v8.pm12374452

[111] Bolinger A, Zangwill A, Slattery J, Glidden D, DeSantes K, Heyn L, et al. An evaluation of engraftment, toxicity and Busulfan concentration in children receiving bone marrow transplantation for leukemia or genetic disease. Bone Marrow Transpl. 2000;25:925–30. 10.1038/sj.bmt.1702371 10800058

[112] Ljungman P, Hassan M, Békássy A, Ringdén O, Öberg G. High Busulfan concentrations are associated with increased transplant-related mortality in allogeneic bone marrow transplant patients. Bone Marrow Transpl. 1997;20:909–13. 10.1038/sj.bmt.1700994 9422468

[113] Saif MW, Choma A, Salamone SJ, Chu E. Pharmacokinetically guided dose adjustment of 5-Fluorouracil: A rational approach to improving therapeutic outcomes. JNCI: J Natl Cancer Inst. 2009;101:1543–52. 19841331 10.1093/jnci/djp328

[114] Milano G, Etienne MC, Cassuto-Viguier E, Thyss A, Santini J, Frenay M, et al. Influence of sex and age on fluorouracil clearance. J Clin Oncol. 1992;10:1171–5. 1607921 10.1200/JCO.1992.10.7.1171

[115] Gamelin E, Boisdron-Celle M, Guérin-Meyer V, Delva R, Lortholary A, Genevieve F, et al. Correlation between uracil and dihydrouracil plasma ratio, fluorouracil (5-FU) Pharmacokinetic parameters, and tolerance in patients with advanced colorectal cancer: A potential interest for predicting 5-FU toxicity and determining optimal 5-FU dosage. J Clin Oncol. 1999;17:1105–1105. 10561167 10.1200/JCO.1999.17.4.1105

[116] Gamelin E, Delva R, Jacob J, Merrouche Y, Raoul JL, Pezet D, et al. Individual fluorouracil dose adjustment based on Pharmacokinetic Follow-Up compared with conventional dosage: results of a multicenter randomized trial of patients with metastatic colorectal cancer. J Clin Oncol. 2008;26:2099–105. 18445839 10.1200/JCO.2007.13.3934

[117] Greibe E, Sorensen B, Meldgaard P, Hoffmann-Lücke E. Development and validation of an LC-MS/MS method for quantification of osimertinib and its two metabolites AZ7550 and AZ5104 in human plasma including long-time storage. J Pharm Biomed Anal. 2025;255:116662. 39787847 10.1016/j.jpba.2025.116662

[118] Kapoor A, Figlin RA. Targeted Inhibition of mammalian target of Rapamycin for the treatment of advanced renal cell carcinoma. Cancer. 2009;115:3618–30. 19479976 10.1002/cncr.24409

[119] MacDonald A, Scarola J, Burke JT, Zimmerman JJ. Clinical pharmacokinetics and therapeutic drug monitoring of sirolimus. Clin Ther. 2000;22:B101–21. 10823378 10.1016/s0149-2918(00)89027-x

[120] Marty FM, Lowry CM, Cutler CS, Campbell BJ, Fiumara K, Baden LR, et al. Voriconazole and sirolimus coadministration after allogeneic hematopoietic stem cell transplantation. Biol Blood Marrow Transplant. 2006;12:552–9. 16635790 10.1016/j.bbmt.2005.12.032

[121] Cattaneo D, Cortinovis M, Baldelli S, Gotti E, Remuzzi G, Perico N. Limited sampling strategies for the Estimation of sirolimus daily exposure in kidney transplant recipients on a calcineurin Inhibitor—Free regimen. J Clin Pharmacol. 2009;49:773–81. 19491334 10.1177/0091270009332811

[122] Sánchez-Fructuoso AI. Everolimus: an update on the mechanism of action, pharmacokinetics and recent clinical trials. Expert Opin Drug Metab Toxicol. 2008;4:807–19. 18611120 10.1517/17425255.4.6.807

[123] Joerger M, Schellens JH, Beijnen JH. Therapeutic drug monitoring of non-anticancer drugs in cancer patients. Methods Find Exp Clin Pharmacol. 2004;26:531. 15538543 10.1358/mf.2004.26.7.863736

[124] le Coutre P, Kreuzer K-A, Pursche S, v. Bonin M, Leopold T, Baskaynak G, et al. Pharmacokinetics and cellular uptake of Imatinib and its main metabolite CGP74588. Cancer Chemother Pharmacol. 2004;53:313–23. 14658008 10.1007/s00280-003-0741-6

[125] Gao B, Yeap S, Clements A, Balakrishnar B, Wong M, Gurney H. Evidence for therapeutic drug monitoring of targeted anticancer therapies. J Clin Oncol. 2012;30:4017–25. 22927532 10.1200/JCO.2012.43.5362

[126] Hudes G, Carducci M, Tomczak P, Dutcher J, Figlin R, Kapoor A, et al. Temsirolimus, interferon alfa, or both for advanced Renal-Cell carcinoma. N Engl J Med. 2007;356:2271–81. 17538086 10.1056/NEJMoa066838

[127] Partridge AH. Adherence to therapy with oral antineoplastic agents. CancerSpectrum Knowl Environ. 2002;94:652–61. 10.1093/jnci/94.9.652 11983753

[128] Marin D, Bazeos A, Mahon F-X, Eliasson L, Milojkovic D, Bua M, et al. Adherence is the critical factor for achieving molecular responses in patients with chronic myeloid leukemia who achieve complete cytogenetic responses on Imatinib. J Clin Oncol. 2010;28:2381–8. 20385986 10.1200/JCO.2009.26.3087 PMC6366340

[129] van Erp NP, Gelderblom H, Guchelaar H-J. Clinical pharmacokinetics of tyrosine kinase inhibitors. Cancer Treat Rev. 2009;35:692–706. 19733976 10.1016/j.ctrv.2009.08.004

[130] Menz BD, Stocker SL, Verougstraete N, Kocic D, Galettis P, Stove CP, et al. Barriers and opportunities for the clinical implementation of therapeutic drug monitoring in oncology. Br J Clin Pharmacol. 2021;87:227–36. 32430968 10.1111/bcp.14372

[131] Vithanachchi DT, Maujean A, Downes MJ, Scuffham P. A comprehensive review of economic evaluations of therapeutic drug monitoring interventions for cancer treatments. Br J Clin Pharmacol. 2021;87:271–83. 32692416 10.1111/bcp.14494

[132] Erku D, Schneider J, Scuffham P. A framework for economic evaluation of therapeutic drug monitoring—guided dosing in oncology. Pharmacol Res Perspect. 2021;9. 10.1002/prp2.862 PMC8453491 34546005

[133] Skirvin JA, Lichtman SM. Pharmacokinetic considerations of oral chemotherapy in elderly patients with cancer. Drugs Aging. 2002;19:25–42. 11929325 10.2165/00002512-200219010-00003

[134] Lee JJ, Beumer JH, Chu E. Therapeutic drug monitoring of 5-fluorouracil. Cancer Chemother Pharmacol. 2016;78:447–64. 27217046 10.1007/s00280-016-3054-2 PMC5204259

[135] Wilhelm M, Mueller L, Miller MC, Link K, Holdenrieder S, Bertsch T, et al. Prospective, multicenter study of 5-Fluorouracil therapeutic drug monitoring in metastatic colorectal cancer treated in routine clinical practice. Clin Colorectal Cancer. 2016;15:381–8. 27256667 10.1016/j.clcc.2016.04.001

[136] Fleisher B, Ait-Oudhia S. A retrospective examination of the US food and drug administration’s clinical Pharmacology reviews of oncology biologics for potential use of therapeutic drug monitoring. Onco Targets Ther. 2017;11:113–21. 29343970 10.2147/OTT.S153056 PMC5749565

[137] Oude Munnink T, Henstra M, Segerink L, Movig K, Brummelhuis-Visser P. Therapeutic drug monitoring of monoclonal antibodies in inflammatory and malignant disease: translating TNF‐α experience to oncology. Clin Pharmacol Ther. 2016;99:419–31. 26265133 10.1002/cpt.211

[138] Syversen SW, Jørgensen KK, Goll GL, Brun MK, Sandanger Ø, Bjørlykke KH, et al. Effect of therapeutic drug monitoring vs standard therapy during maintenance Infliximab therapy on disease control in patients with Immune-Mediated inflammatory diseases. JAMA. 2021;326:2375. 34932077 10.1001/jama.2021.21316 PMC8693274

[139] Patnaik A, Kang SP, Rasco D, Papadopoulos KP, Elassaiss-Schaap J, Beeram M, et al. Phase I study of pembrolizumab (MK-3475; Anti–PD-1 monoclonal Antibody) in patients with advanced solid tumors. Clin Cancer Res. 2015;21:4286–93. 25977344 10.1158/1078-0432.CCR-14-2607

[140] Vaes M, Hites M, Cotton F, Bourguignon AM, Csergö M, Rasson C, et al. Therapeutic drug monitoring of posaconazole in patients with acute myeloid leukemia or myelodysplastic syndrome. Antimicrob Agents Chemother. 2012;56:6298–303. 23027198 10.1128/AAC.01177-12 PMC3497193

[141] Chevreau C, Massard C, Flechon A, Delva R, Gravis G, Lotz J, et al. Multicentric phase II trial of TI-CE high‐dose chemotherapy with therapeutic drug monitoring of carboplatin in patients with relapsed advanced germ cell tumors. Cancer Med. 2021;10:2250–8. 33675184 10.1002/cam4.3687 PMC7982623

[142] de Wit D, van Erp NP, den Hartigh J, Wolterbeek R, den Hollander-van Deursen M, Labots M, et al. Therapeutic drug monitoring to individualize the dosing of pazopanib. Ther Drug Monit. 2015;37:331–8. 25271729 10.1097/FTD.0000000000000141

[143] Cuocolo R, Caruso M, Perillo T, Ugga L, Petretta M. Machine learning in oncology: A clinical appraisal. Cancer Lett. 2020;481:55–62. 32251707 10.1016/j.canlet.2020.03.032

[144] Shreve JT, Khanani SA, Haddad TC. Artificial intelligence in oncology: current capabilities, future opportunities, and ethical considerations. Am Soc Clin Oncol Educational Book. 2022;:842–51. 10.1200/EDBK_350652 35687826

[145] Liang G, Fan W, Luo H, Zhu X. The emerging roles of artificial intelligence in cancer drug development and precision therapy. Biomed Pharmacother. 2020;128:110255. 32446113 10.1016/j.biopha.2020.110255

[146] Kang J, Chowdhry AK, Pugh SL, Park JH. Integrating artificial intelligence and machine learning into cancer clinical trials. Semin Radiat Oncol. 2023;33:386–94. 37684068 10.1016/j.semradonc.2023.06.004 PMC10880815

[147] Athanasopoulou K, Daneva G, Boti M, Dimitroulis G, Adamopoulos P, Scorilas A. The transition from cancer omics to epi-omics through Next- and Third-Generation sequencing. Life. 2022;12:2010. 36556377 10.3390/life12122010 PMC9785810

[148] Srivastava R. Role of transcriptomics in precision oncology. Rep Radiotherapy Oncol. 2024;11.

[149] Hussen BM, Abdullah ST, Salihi A, Sabir DK, Sidiq KR, Rasul MF, et al. The emerging roles of NGS in clinical oncology and personalized medicine. Pathol Res Pract. 2022;230:153760. 35033746 10.1016/j.prp.2022.153760

[150] Briki M, André P, Thoma Y, Widmer N, Wagner AD, Decosterd LA, et al. Precision oncology by Point-of-Care therapeutic drug monitoring and dosage adjustment of conventional cytotoxic chemotherapies: A perspective. Pharmaceutics. 2023;15:1283. 37111768 10.3390/pharmaceutics15041283 PMC10147065

[151] Gaspar V, Ibrahim S, Zahedi R, Borchers C. Utility, promise, and limitations of liquid chromatography-mass spectrometry‐based therapeutic drug monitoring in precision medicine by Vanessa P. Gaspar, Sahar ibrahim, René P. Zahedi and Christoph H. Borchers. J Mass Spectrom. 2021;56. 10.1002/jms.4788 PMC8597589 34738286

